# The Chelating Abilities of Tertiary Amines with N-O-Donors Towards Cu(II) Ions and the Catalytic Properties of the Resulting Complexes

**DOI:** 10.3390/molecules30163419

**Published:** 2025-08-19

**Authors:** Martina Zonzin, Martina Chianese, Andrea Squarcina, Degnet Melese Dereje, Ambra Campofelice, Alessia Da Fermo, Federica Belluti, Nadia Marino, Filip Dębicki, Aleksandra Kotynia, Aleksandra Marciniak, Justyna Brasuń, Mauro Carraro

**Affiliations:** 1 Department of Chemical Sciences, University of Padova, Via F. Marzolo 1, 35131 Padova, Italy; 2 Department of Chemistry, Ludwig-Maximilians Universität (LMU), Butenandtstr, 5-13, 81377 München, Germany; 3 Department of Pharmacy and Biotechnology, University of Bologna, Via Belmeloro 6, 40126 Bologna, Italy; 4 Department of Chemistry and Chemical Technologies, University of Calabria, Via P. Bucci 14/c, 87036 Arcavacata Di Rende, CS, Italy; 5 4^TH^ Military Clinical Hospital, Weigla 5, 53-114 Wroclaw, Poland; 6 Department of the Basic Chemical Sciences, Wroclaw Medical University, 50-556 Wroclaw, Poland; 7 ITM-CNR UoS of Padova, Via F. Marzolo 1, 35131 Padova, Italy

**Keywords:** copper complexes, N_3_O donors, antioxidants, chelators, amyloid aggregation

## Abstract

Oxidative stress, driven by excess reactive oxygen species (ROS), is a key factor in the progression of neurodegenerative diseases like Alzheimer’s disease (AD). In this context, copper dysregulation can also contribute to this imbalance, being responsible for enhanced ROS production, so that copper scavenging has been investigated as a possible therapeutic strategy. This study investigates the behavior of two isostructural ligands, featuring an N_3_O donor set, that effectively chelate Cu(II) in aqueous solution. Interestingly, their resulting mono- or dinuclear copper complexes feature a coordination environment suitable to foster antioxidant activity. By transforming copper’s oxidant potential into antioxidant action, these systems may reduce copper-induced oxidative damage. The work examines the pH-dependent metal-binding behavior of the ligands, the catalytic properties of the resulting complexes under physiological conditions, and their ability to inhibit β-amyloid peptide aggregation.

## 1. Introduction

Superoxide anion, hydrogen peroxide, and hydroxyl radical are defined as Reactive Oxygen Species (ROS), known for causing rapid, short-range, and non-selective oxidative transformations, leading to cellular oxidative stress [1]. A potential solution to reduce oxidative damage is the use of antioxidants. Among the most important natural antioxidants are the enzymes superoxide dismutase (SOD) and catalase (CAT), which dismutate superoxide radicals and hydrogen peroxide, respectively. The most common natural SODs are MnSOD and Cu-ZnSOD. MnSODs are found as tetramers in the mitochondrial matrix and as dimers in bacterial cytoplasm, while Cu-ZnSODs are mainly cytosolic and contain a dinuclear copper–zinc core [2,3]. Structurally, both enzymes are rich in N donors (histidine residues) and include O donors (acidic residues and water), completing the coordination sphere and fine-tuning the Cu(II)/Cu(I) and Mn(III)/Mn(II) redox potentials necessary for the dismutation reactions [4]. Two distinct classes of naturally occurring catalases are known: while iron catalases are tetramers of identical subunits, each containing a ferriprotoporphyrin as a prosthetic group [5], MnCAT’s active site includes two manganese atoms bridged by a glutamate carboxylate and by two water-derived ligands. Each manganese center is also coordinated by one glutamate and one histidine [6].

From a therapeutic standpoint, natural CATs and SODs face limitations in “antioxidant therapy” because of limited hydrolytic stability and high synthetic cost [1]. Consequently, nutritional antioxidants (vitamins C, polyphenols, and β-carotene) are currently the preferred choice [7]. An appealing alternative lies in the use of catalytic antioxidants that mimic natural enzymes (biomimetics) like SOD and CAT. These metal-based complexes, often involving manganese, iron, or copper, can detoxify ROS species efficiently at low concentrations and with sustained activity [8,9]. Significant research efforts were made to develop synthetic biomimetics with combined SOD-CAT activity, which cannot be achieved by natural enzymes due to their specificity [10,11]. Yet, major challenges remain, including poor solubility and stability of such complexes in aqueous environments, often leading to unintended release of metal ions. Additionally, many complexes exhibit significant undesirable oxidant activity [12,13,14,15,16,17,18,19,20,21,22].

Within this scenario, copper ions play a multifaceted role, functioning not only as active sites in superoxide dismutase (SOD) enzymes but also contributing to metal-mediated oxidative stress associated with neurodegenerative diseases such as Alzheimer’s disease (AD), where they further promote the aggregation of β-amyloid peptides into toxic oligomers [23]. In AD patients, indeed, copper dyshomeostasis results in significantly increased extracellular and reduced intracellular copper levels. In this case, free copper ions become toxic targets, necessitating effective strategies—such as Cu(II) chelation—to stabilize the metal, limit redox cycling, and mitigate oxidative damage [24,25,26,27]. 

**Scheme 1:**
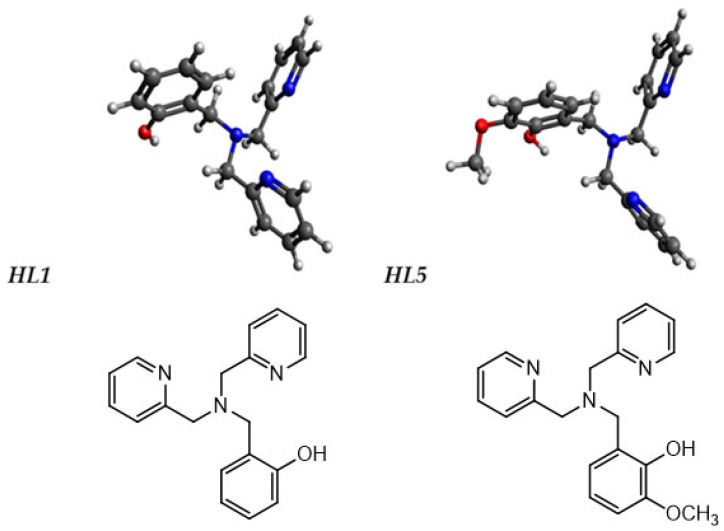
The schematic structures of the HL1 and HL5 ligands at pH 7.4 were obtained with the Avogadro software, Application Version: 1.2.0 [30]. Color code for the atoms: O—red, N—blue, H—white, C—black.

In a previous paper, three isostructural N_6_O-type ligands were proposed to form dinuclear Cu complexes in situ, providing a suitable coordination environment to convert the oxidant activity of copper ions into a beneficial antioxidant activity [28]. Following a similar approach, the present work exploits simpler N_3_O-type ligands to prepare Cu complexes with dual SOD/CAT activity. The HL1 ligand is a tertiary amine, N-(2-hydroxylbenzyl)-N,N-bis(2-pyridylmethyl)amine, bearing two pyridines and a phenol residue separated from the nitrogen by a single methylene group, while in HL5, 2-{[Bis(pyridin-2-ylmethyl)amino]methyl}-6-methoxyphenol, the meta position of the phenol ring is functionalized with a methoxy group (Scheme 1) [29].

These ligands offer several advantages for a multitarget therapy [31]. They are multidentate, containing at least four nitrogen and oxygen donor atoms, allowing for versatile coordination and formation of stable chelate complexes. This structural feature also mimics natural metal-binding motifs found in metalloenzymes. The phenol group, indeed, can deprotonate under slightly basic conditions to form a phenoxide anion (-O^−^), a strong donor that enhances metal binding. Moreover, the geometry and flexibility of these ligands are well-suited to match the preferred coordination geometries of Cu(II), such as distorted square planar or square pyramidal arrangements. These characteristics contribute to the stability and tunability of the resulting complexes, making them attractive for the development of redox-active catalysts. Moreover, in HL5, the methoxy (-OCH_3_) group might (i) increase the electron density at the copper center, (ii) serve as an additional oxygen donor, and (iii) provide better solubility in aqueous environments.

This research work focuses on the coordinating and antiaggregating abilities of the free ligands and on the catalytic activity of the corresponding copper complexes. In particular, the chelation ability of the ligands was explored in an aqueous environment, and the resulting complexes were the object of potentiometric solution speciation, cyclic voltammetry analysis, and catalytic tests for monitoring both antioxidant and oxidant activities. Finally, their interaction with β-amyloid peptides was examined.

## 2. Results and Discussion

### 2.1. Coordination Studies

The two ligands, HL1 and HL5, were synthesized, characterized, and employed in this study to obtain copper complexes. Although dimerization to form 2:2 complexes cannot be entirely ruled out, potentiometric data did not support the formation of dinuclear species in situ, as their inclusion worsened the fitting. Therefore, we focused primarily on mononuclear species, which more accurately reflect the solution behavior observed during pH-dependent measurements. 

However, isolated mononuclear and dinuclear complexes were also examined to enable structural comparisons and support catalytic studies under well-defined conditions, where mechanistic insights can be more effectively obtained. Although dimeric complexes apparently do not form in solution under the studied conditions, they were included to provide a broader structural and functional perspective.

The detailed protocols for the synthesis and characterization of the ligands and their Cu(II) complexes are provided in the Supplementary Materials. These includes the ^1^H NMR, ^13^C NMR, and 2D NMR data for the ligands, as well as the crystal structures of the complexes. The complexes were isolated using Cu(ClO_4_)_2_ as the Cu(II) source in the presence of a stoichiometric amount of ligand. Dinuclear complexes were prepared upon addition of NEt_3_.

#### Potentiometric Studies

**Figure 1:**
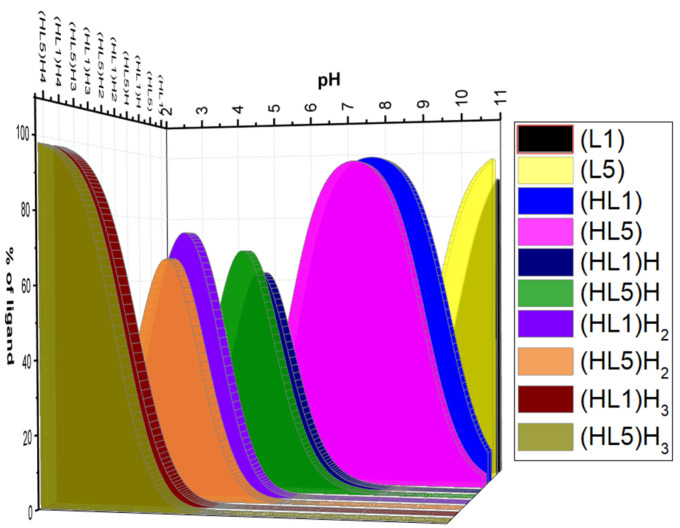
Speciation curves for the ligands HL1 and HL5. Conditions: [ligands] = 0.7 mM in 5% *v*/*v* DMSO/H_2_O (with HCl/KCl, I = 0.1 M; initial pH = 2.7).

Initially, potentiometric measurements were performed to investigate the binding abilities of the ligands in an aqueous environment at different pH levels and to construct the corresponding speciation curves (Figure 1). The analyses were performed in HCl/KCl mixtures, with KCl used to maintain a constant ionic strength of 0.1 mol·dm^−3^. The first step of the potentiometric studies was the assessment of the acid-base properties of both ligands. The overall and stepwise protonation constants are collected in Table 1.

Both ligands are characterized by four protonation constants, whereby the first one is ascribed to the phenolic group. At a physiological pH (6.8–7.8), both molecules display undissociated (-OH) phenolic groups (Figure 1). The next three protonation constants are similar and are related to the protonation of the tertiary nitrogen atoms and of pyridine residues, yielding ammonium ions. The presence of the methoxy substituent in the HL5 molecule decreases all protonation constants.

In the next step, the system was analyzed in the presence of copper ions. UV–Vis titration of the ligand solutions with Cu(II) revealed the formation of 1:1 metal-to-ligand complexes for both ligands (Figures S38–S40, Supplementary Materials). The equimolar 1:1 complexation is also supported by the Job plot, which shows a maximum signal at a molar fraction of *X*_Cu(II)_ = 0.5 (Figure S41, Supplementary Materials). 

**Figure 2:**
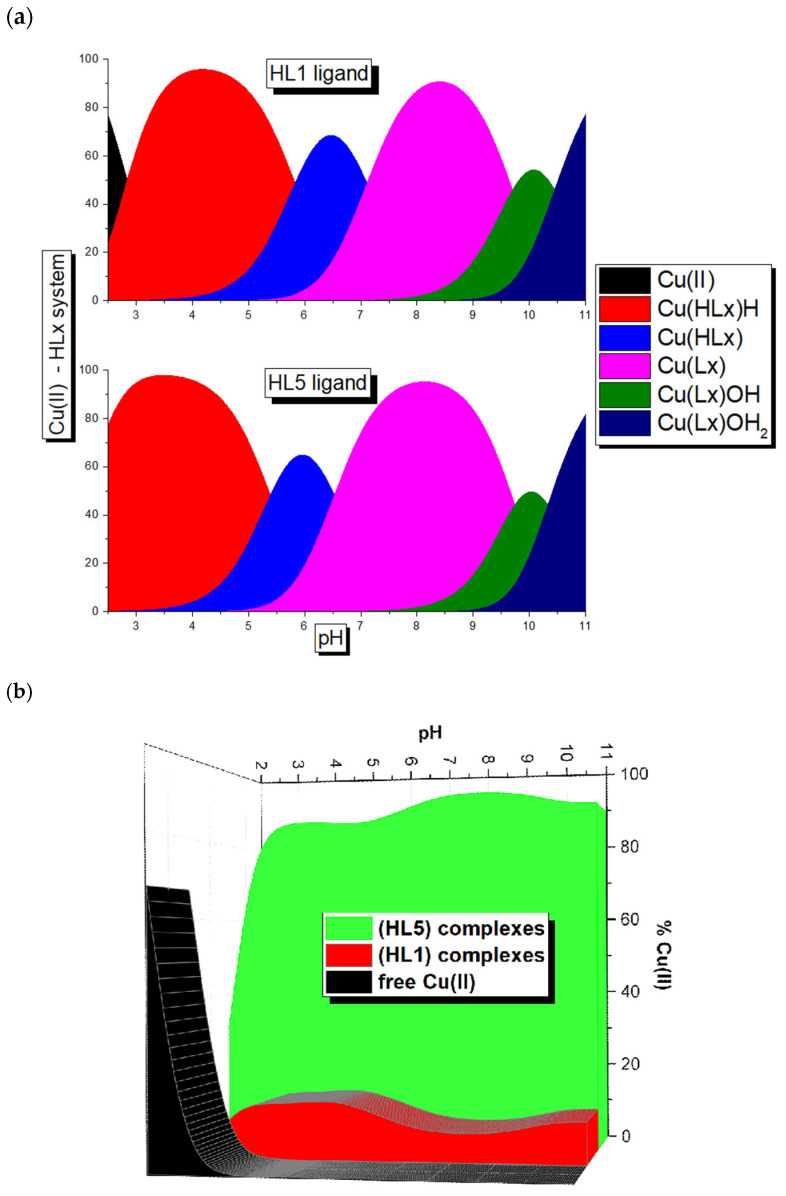
(**a**) Speciation curves for the in situ-formed complexes ligand/copper (II) 1:1. with ligands HL1 and HL5 (0.7 mM) in 5% *v*/*v* DMSO/H_2_O (containing HCl and KCl, I = 0.1 M; initial pH = 2.7). (**b**) The competition diagram for the system HL1:Cu(II):HL5 with molar ratio of 1:1:1.

Based on the potentiometric studies, the overall and stepwise stability constants of formed complexes were obtained and collected in Table 2; the speciation curves of in situ-formed complexes are presented in Figure 2.

In the explored pH range (between 2 and 11), both ligands form five types of complexes, as listed in Table 2. Between pH 3.5 and pH 5.0, the predominant species is the [Cu(HLx)H]^3+^. This complex, formed by the HL5, reaches its maximum concentration at pH 3.5, whereas the complex with the same stoichiometry, formed by HL1, reaches its highest concentration at pH 4.5. The formation of this complex by both ligands arises from the dissociation of two protons of the starting ligand (HLx)H_3_, found at low pH. However, it is difficult to unambiguously define the groups from which protons dissociate (Figure 2a).

**Scheme 2:**
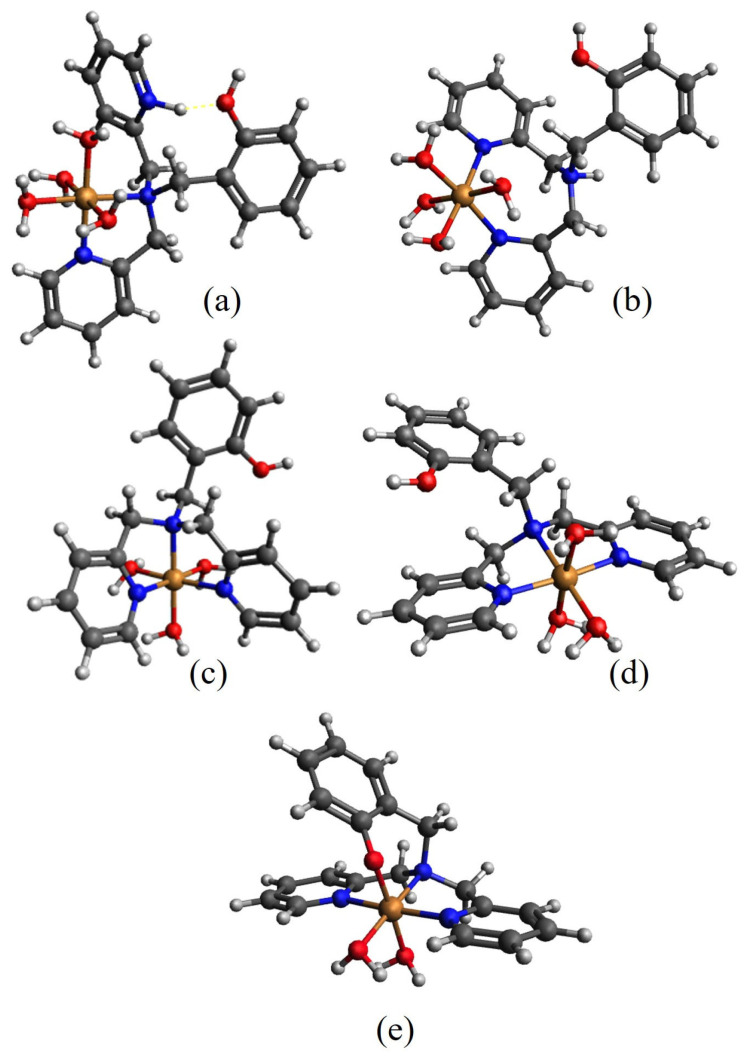
The schematic structures of the [Cu(HLx)H_2_]^3+^ (**a**)—the {N_tertiary amine_, N_Pyr_}, (**b**)—the {2xN_Pyr_} binding modes, (**c**,**d**)—[Cu(HLx)H]^2+^ and (**e**)—[Cu(HLx)]^+^ for HL1, taken as example, obtained with the Avogadro software, Application Version: 1.2.0 [30]. Color code for the atoms: O—red, N—blue, H—white, C—black, Cu—orange.

Scheme 2 illustrates some proposed coordination modes, which serve as representative models of the species present in solution. There are two possibilities to form the [Cu(HLx)H]^3+^ complexes: binding a N_tertiary amine_ and N_Pyr_ or both N_Pyr_ (Scheme 2a,b): the involvement of the N_tertiary amine_ and N_Pyr_ causes the formation of the five-membered chelating ring (Scheme 2a), whilst in the second option a seven-member chelating ring is formed (Scheme 2b). Based on the fact that the five-member chelating ring is more favored and thermodynamically stable [32], it can be assumed that the [Cu(HLx)H]^3+^ complex is characterized by the {N_tertiary amine_, N_Pyr_} binding mode.

Upon pH increase, the formation of two complexes: [Cu(HLx)]^2+^, [Cu(Lx)]^+^ occurs. The stepwise stability constant for the first one is p*K* = 5.83 and 5.39 (for HL1 and HL5, respectively, Table 2), corresponding to the equilibrium:

[Cu(HLx)H]^3+^ ⇄ [Cu(HLx)]^2+^ + H^+^

**Figure 3:**
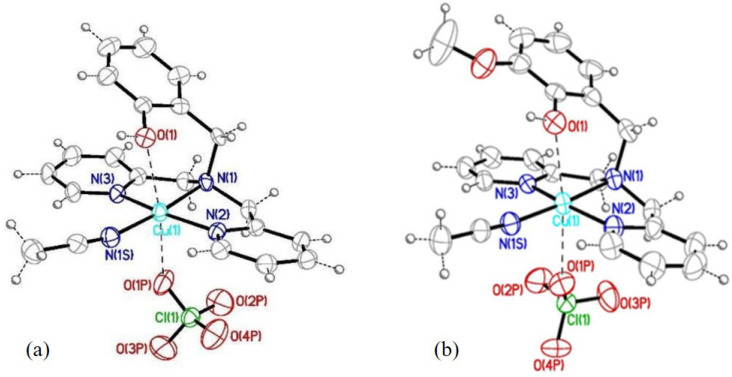
Molecular structures of (**a**) [Cu(HL1)(CH_3_CN)ClO_4_]^+^ and (**b**) [Cu(HL5)(CH_3_CN)ClO_4_]^+^ cations with selected atomic labels (ellipsoids drawn at the 30% probability level). See Tables S1–S3 in the Supplementary Materials for details.

This step likely reflects the coordination of a third nitrogen donor—specifically, the second pyridyl (N_Pyr_) group—to the Cu(II) ion. Two plausible coordination geometries can be proposed for this species: one in which the tertiary amine nitrogen occupies an axial position (Scheme 2c) and another where it is located equatorially (Scheme 2d). The X-ray crystallographic studies indicate that the latter arrangement is the most likely one, being also observed for both complexes isolated in CH_3_CN in the presence of ClO_4_^−^ anions (Figure 3, Figures S26 and S34). The next mononuclear complex [Cu(Lx)]^+^ appears in both systems with the highest concentration around pH 8, and in the system with HL5, it prevails in a wider pH range. The formation of this species is related to the involvement of the oxygen donor, O*_Phe_*, of the phenolate residue and the formation of the N_3_O-type complex. In the absorption spectra recorded above pH 5.5–6.0 for both ligands, an additional band appears around 450 nm. Based on literature data, this feature can be attributed to a ligand-to-metal charge transfer (LMCT) transition (Figure S42a,b) [33,34]. Moreover, consistent with the previously discussed results, the discussed complex is characterized by the {{N_tertiary amine_, 2xN_Pyr_}{O_Phe_}_axial_} coordination manner.

Above pH 9, the formation of the last two species, [Cu(Lx)OH] and [Cu(Lx)OH_2_]^−^, may be ascribed to the loss of the protons from the first and second water molecules bound to the Cu(II) ion [35]. The appearance of these complexes influences the spectral features of the investigated system (Figure S42a,b). It is also worth noting that at high pH, these complexes are known to release the metal ion.

These data suggest that at physiological pH the equilibrium involving the deprotonation of the ligand results in the formation of the corresponding mononuclear complexes [Cu(HLx)]^2+^ and [Cu(Lx)]^+^.

Despite the similar coordinating behavior of both ligands, their binding abilities toward copper ions are influenced by the absence (HL1)/presence (HL5) of the methoxy group on the phenolic ring. Indeed, the competition diagram for the HL1:Cu(II):HL5 system (molar ratio 1:1:1, Figure 2b) shows that HL1 without the methoxy substituent on the phenolic ring is significantly less efficient in metal ion binding between pH 3 and 11.

### 2.2. Catalytic Ability

To evaluate the catalytic potential, the behavior of the complexes formed in situ was compared with that of the complexes isolated, upon crystallization, from organic solvents (CH_3_CN or CH_3_OH) using Cu(ClO_4_)_2_ as the Cu(II) source, in the presence of a stoichiometric amount of the ligands (see the experimental part and Supplementary Materials).

To assess the antioxidant capabilities of the complexes, their electron transfer properties were first analyzed. Following this, the superoxide dismutase-like and catalase-like activities of the complexes were investigated, aiming to evaluate their potential to mimic enzymatic defense mechanisms against ROS. Finally, the oxidant activity was evaluated through the use of two model substrates.

#### 2.2.1. Cyclic Voltammetry

For the complexes to exhibit superoxide dismutase activity, the copper center must efficiently catalyze superoxide (O_2_^−^•) disproportionation via rapid and reversible Cu(II)/Cu(I) redox cycling. To assess this behavior, the corresponding redox potentials were measured by cyclic voltammetry (CV), offering key insights into the electron-transfer properties of the complexes. The metal ion redox potential should ideally lie between the potential for oxidation (−0.16 V vs. NHE) (Equation (1)) and reduction (0.89 V vs. NHE) of superoxide radicals (Equation (2)).

(1) $$O_{2}^{∙-}\to O_{2}+e^{-}$$

(2) $$O_{2}^{∙-}+e^{-}+H^{+}\to H_{2}O_{2}$$

**Figure 4:**
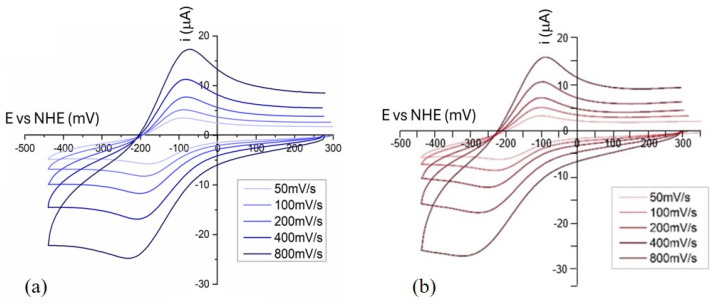
Cyclic voltammograms of the complex (**a**) Cu(HL1)(ClO_4_)_2_ and (**b**) Cu(HL5)(ClO_4_)_2_: recorded at scan rates of 50 mV/s, 100 mV/s, 200 mV/s, 400 mV/s, 800 mV/s. Complex = 0.50 mM in 5% *v*/*v* CH_3_CN/H_2_O (10 mM phosphate buffer, 100 mM NaCl, pH 7.8).

Figure 3 shows cyclic voltammograms, obtained at different scan rates, of the mononuclear complexes Cu(HL1)(ClO_4_)_2_ and Cu(HL5)(ClO_4_)_2_, whilst Figure S43a,b shows those of the dinuclear complexes Cu_2_L1_2_(ClO_4_)_2_ and Cu_2_L5_2_(ClO_4_)_2,_ each one compared with complexes obtained in situ. The key electrochemical parameters extracted from the cyclic voltammograms are summarized in Table 3, highlighting a similar redox behavior of the complexes with both ligands. However, the nature of the anion appears to influence the properties of the mononuclear species formed in situ, compared with the isolated complexes. Notably, the E_1_/_2_ values [calculated as $E_{1/2}=(E_{pc}+E_{pa})/2$] fall within the optimal electrochemical window for superoxide dismutation only in the case of the mononuclear complexes (Figure 4).

All curves show the mono-electronic transfer with a quasi-reversible character (Table 3), with |ΔE| = E_pc_ − E_pa_ values higher than 59 mV.

**Figure 5:**
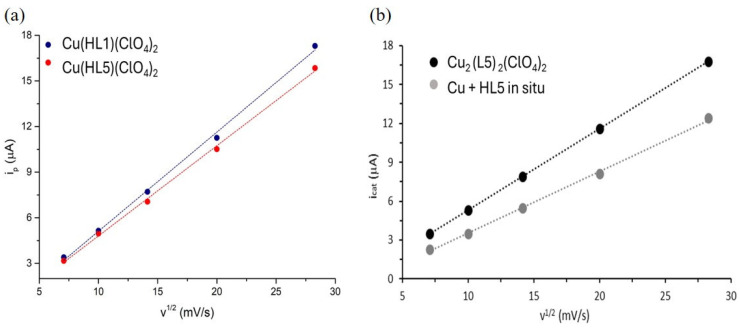
Graph “*i_p_ vs.* ${\sqrt{v}}_{scan\;}$” obtained from the cyclic voltammograms of the complexes: (**a**) Cu(HL1)(ClO_4_)_2_ and Cu(HL5)(ClO_4_)_2_; (**b**) Cu_2_L5_2_(ClO_4_)_2_ preformed and mononuclear complex formed in situ. The graphs were recorded at the scan rates of 50, 100, 200, 400, and 800 mV/s.

The reversibility of the mono-electronic transfer was also confirmed by the plot “*i_p_ (μA) vs.* ${\sqrt{v}}_{scan\;}(mV/s)”$, (Figure 5a,b), which shows for each complex (both mononuclear, as well as the HL5 complex produced in situ and its preformed dinuclear complex) a linear trend, consistent with the Randles–Sevcik equation for a reversible process. Nevertheless, for the mononuclear complexes, the (i_pa_/i_pc_) peak current ratio is below unity, likely resulting from alterations in the metal center’s coordination environment during electron transfer. Such behavior has been observed in recent studies, where alterations in the coordination sphere during electron transfer processes have been linked to deviations in peak current ratios, indicating structural adjustments that hinder back electron transfer [36].

#### 2.2.2. SOD Activity

Besides redox potential, SOD activity strongly depends on other properties [34]. The ligand framework plays a crucial role in this context, as it must exhibit strong affinity for both oxidation states of the metal to ensure complex stability under physiological conditions and to prevent metal ion release during redox cycling [37]. In addition, an accessible site for binding O_2_^•−^ is essential to enable inner-sphere electron transfer, facilitating efficient dismutation and minimizing off-pathway reactions [3].

Signorella et al. (2025) [38] reported that copper complexes with more flexible ligands exhibit higher catalytic rates for superoxide dismutation, even when their redox potential is less favorable. This highlights how ligand flexibility facilitates the necessary geometric rearrangements during redox cycling, thereby improving catalytic efficiency. The mononuclear complexes appear to meet these criteria effectively. The N_3_O donor set, formed by the pyridines and tertiary amine, mimics the histidine residues structurally, while the phenol group serves as a hydrogen bond donor/acceptor. Moreover, the ligands’ conformational adaptability enables them to accommodate the geometric transition from Cu(II) (square planar) to Cu(I) (distorted tetrahedral), which is essential for superoxide binding and efficient electron transfer.

To reduce uncertainty about the actual active species involved in the reaction, the catalytic investigations were made with the isolated species. The ability to catalyze superoxide dismutation was assessed using the indirect cytochrome C-based assay originally introduced by McCord and Fridovich [39]. In this kind of test, the presence of a catalyst with SOD activity, acting as a scavenger for O_2_^•−^ and generated via a xanthine/xanthine oxidase system, prevents the electron transfer to cytochrome C (Cyt C) to form ferricytochrome. By measuring the absorbance associated with ferricytochrome (λ = 550 nm) using UV–Vis and reporting it as a function of time, the IC_50_ value (the concentration of the catalyst with SOD activity that causes 50% suppression of cytochrome C reduction) and the catalytic constant (Equation (3)) can be obtained.

(3) $$k_{cat}(O_{2}^{∙-})=k_{CytC}\cdot \frac{\left[CytC\right]}{{IC}_{50}},\;\mathrm{where}\;k_{\mathrm{CytC}}=2.6\times 10^{5}\;M^{-1}s^{-1}$$

Comparing the IC_50_ values and the *k_cat_* of the synthesized complexes with the values collected in Table 4, it is possible to notice that the investigated compounds exhibit lower activity compared with native Cu-ZnSOD as well as to other Cu(II) complexes documented in the literature. However, for all investigated systems, the elimination of O_2_^−^ proceeds with a rate constant approximately two orders of magnitude greater than that of the spontaneous self-dismutation (8 × 10^4^ M^−1^s^−1^ [40]).

The synthesized mononuclear complexes of HL1 and HL5 have slightly better SOD-like activity than the corresponding dimeric complexes: in fact, the IC_50_ values for Cu(HL1)(ClO_4_)_2_ and Cu(HL5)(ClO_4_)_2_ are lower than those of Cu_2_(L1)_2_(ClO_4_)_2_ and Cu_2_(L5)_2_(ClO_4_)_2_, respectively. Besides confirming the observed redox trend, this suggests, on one hand, that the dimeric complexes do not readily dissociate into monomeric species in aqueous solution, and on the other hand, that the active site of the dinuclear complexes is likely less accessible for binding the superoxide radical anion. Among the complexes studied in this work, the one that presents the best SOD-like activity is Cu(HL5)(ClO_4_)_2,_ underscoring its efficiency in removing superoxide radical anions.

#### 2.2.3. CAT Activity

Catalases catalyze the disproportionation of H_2_O_2_ into water and molecular oxygen with remarkable efficiency, exhibiting characteristic *k_cat_*/K_M_ values in the range of 10^6^–10^7^ M^−1^s^−1^, and notably, without the need for external electron-donor substrates [48]. Among their synthetic counterparts, manganese-based complexes are recognized for their pronounced catalytic performance. Nevertheless, the catalase-like activity of copper-based compounds was also well documented. The mechanistic pathway for H_2_O_2_ decomposition in mononuclear copper complexes was delineated by Ramadan et al. [14], whereas Gao et al. [49] proposed a distinct reaction route applicable to dinuclear copper systems. While dinuclear complexes can facilitate bi-electronic H_2_O_2_ dismutation, mononuclear systems are more likely to proceed via radical-based mechanisms [47].

The studied catalase-like activity of the complexes was evaluated by measuring the pressure resulting from the oxygen produced during the dismutation of the hydrogen peroxide. For this analysis, a borate-based buffer system (BBS 50 mM, pH 7.8) was utilized to avoid the competitive binding of phosphate anions. The observed values related to catalyst performance are reported in Table 5. The observed values related to catalyst performance are reported in Table 5.

**Figure 6:**
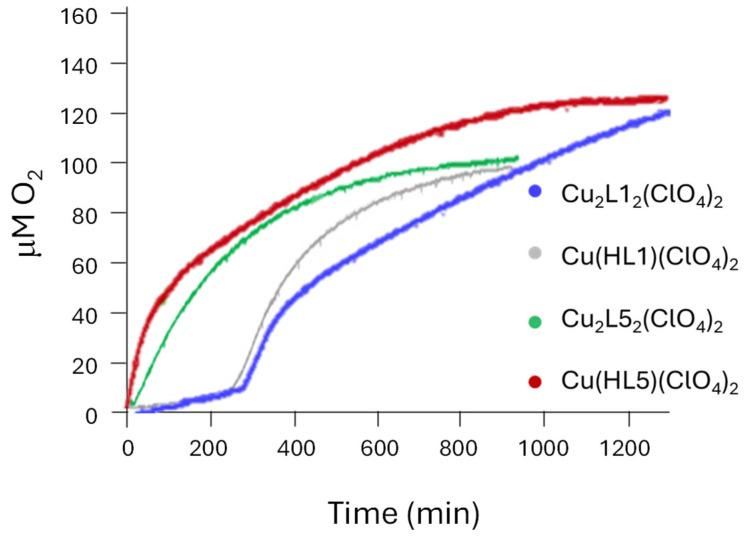
O_2_ production over time, generated from the hydrogen peroxide dismutation, was measured in μmol of O_2_ using pressure data and the ideal gas law.

Examining the graph “*μmol O_2_* vs. *Time*” (Figure 6), it is evident that the complexes reach the maximum O_2_ production in a long time (>900 min).

With respect to other copper complexes reported in the literature, the reactivity appears thus much slower, although with good O_2_ productivity after a prolonged time. Moreover, both HL1 complexes display a significant induction time, probably due to a reorganization of the coordination environment around the copper center that may lead to similar active species. The complexes containing HL5, instead, promptly induce the decomposition of H_2_O_2_. Since induction times may suggest the involvement of a radical initiation step, as in the case of the Fenton reaction, catalase assays were performed in the presence of KBr, which acts as a scavenger for HO• radicals. The addition of KBr significantly enhances the reaction efficiency, as evidenced by the shorter time required to reach the O_2_ production plateau (300–400 min, Figure S44a,b, Supplementary Materials) and the elimination of the induction period observed for the HL1 complexes. These results strongly suggest that radical mechanisms play a crucial role and account for the presence of the initiation step. However, during the prolonged initial lag phase, ligand degradation may occur, leading to copper release. In this context, the increased activity observed with KBr is likely due to a greater stability of the complex.

The reaction progression was monitored by UV–Vis to gather insights into the stability of the complexes during H_2_O_2_ dismutation (Figures S45–S48 in Supplementary Materials). Analyzing the spectra recorded for the systems derived from HL1 and HL5, it is possible to observe changes in the absorption bands. In the case of the mononuclear species containing HL1, the alteration in the absorption band takes place over a comparatively extended period, in agreement with the occurrence of the delay period previously noted. The intensity associated with the π→π * electronic transition progressively diminishes, coinciding with a rise in molecular oxygen generation. Such a trend implies structural reconfiguration within the coordination environment of the complex, which appears necessary to initiate the catalytic event. On the other hand, the characteristic absorption feature vanishes after 4 h as a result of the formation of radical species. The UV–Vis profile of the complex Cu(HL5)(ClO_4_)_2_ (Figure S46) undergoes a faster modification. The π→π * absorbance feature fades within approximately 60 min, and as such, this complex appears less stable than that of HL1 and is likely the reason for the absence of the induction time.

For Cu_2_(L1)_2_(ClO_4_)_2_, the spectrum shows a slight and gradual decrease in the absorbance of the band relative to the transitions π→π *, without abrupt changes at the beginning of the development of O_2_. This behavior suggests that the initial complex slowly converts to the active species. In the case of Cu_2_(L5)_2_(ClO_4_)_2,_ these changes are faster, occurring in the first 90 min; that is, the period of highest speed of the reaction, confirming that the initial complex is quickly rearranged during the reaction.

#### 2.2.4. Peroxidase Activity

**Figure 7:**
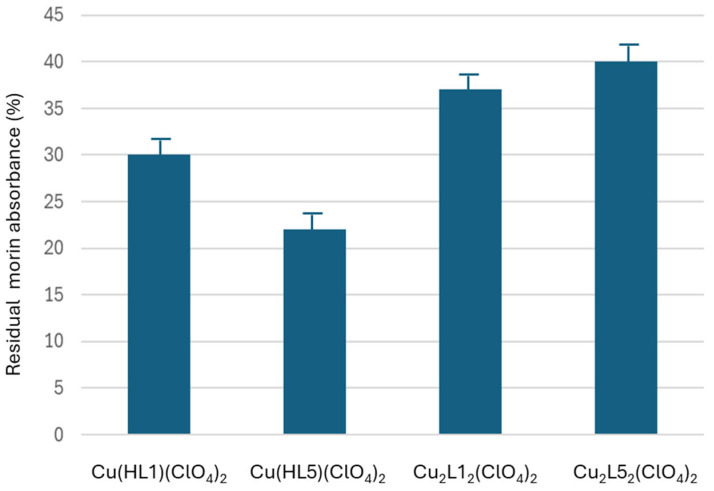
Residual absorbance of morin at the maximum absorption (390 nm) after reaction of the complexes with H_2_O_2_ for 16 h. [Morin] = 0.16 mM, [Catalyst] = 1.6 μM, [H_2_O_2_] = 0.01 M, PBS buffer (50 mM), pH 7.8.

Since initial antioxidant evaluations were conducted without incorporating oxidizable substrates, it is therefore essential to assess the behavior of the complexes in the presence of organic substrates. To this aim, we employed two distinct categories of model substrate: morin and o-phenylenediamine (OPD). We evaluated the spectral changes during morin oxidation, i.e., absorbance decreases for the substrate (Figure 7 and Figures S50–S53 in the Supporting Materials) and increase for the 2,3-diaminephenazine (DAP), the product of the oxidative degradation of the OPD (Figures S55–S58 in the Supporting Materials).

Plotting the absorbance of the morin over time, it is possible to observe that the complexes with the lower peroxidase-like activity are the dinuclear ones (with HL5 displaying 40% residual absorbance after 16 h), while the mononuclear ones are more active (with HL5 giving 21% residual absorbance). Within this scenario, the results obtained for the mononuclear compounds are in agreement with a preferential occurrence of the Fenton reaction.

**Figure 8:**
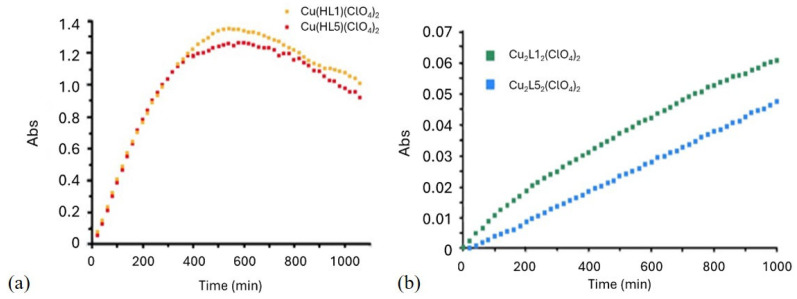
Trend over time at maximum absorption of DAP at 418 nm, with (**a**) Cu(HL1)(ClO_4_)_2_ (yellow) and Cu(HL5)(ClO_4_)_2_ (red), (**b**) Cu_2_L1_2_(ClO_4_)_2_ (blue) and Cu_2_L5_2_(ClO_4_)_2_ (green). [OPD] = 0.32 mM, [Catalyst] = 0.16 mM, [H_2_O_2_] = 0.024 M, PBS buffer (50 mM), pH 7.8.

Concerning the analysis of the oxidant activity with OPD as the substrate, it can be noticed that in the case of the monomeric species, the signal associated with DAP formation reaches a maximum at around 7 h, followed by a decrease due to the overoxidation of OPD (Figure 8a). For the dimeric complexes (Figure 8b), the increase is steady and slower (in the longer timeframe explored, 18 h), confirming the lower oxidant activity for the dimeric Cu complexes, especially with HL5.

### 2.3. Antiaggregation Test

As previously mentioned, Cu(II) ions are involved in neurodegenerative disorders, such as Alzheimer’s disease (AD). In this pathology, a major event is the conversion of soluble Aβ into toxic oligomers [52], which can include Cu ions and further boost the production of ROS, resulting in the oxidative damage of neuronal lipids and proteins [53]. To prevent amyloid aggregation, the key challenge lies in designing molecules that both disrupt β-sheet stacking and block copper ion binding at the metal coordination site located in the N-terminal region of Aβ peptides [54]. For the aggregation test, the Aβ40 peptide was used because it is more soluble than the Aβ42 and it causes a slower aggregation. These tests were performed using the Thioflavin T (Tht) fluorescence method. When amyloid peptide (P) aggregates, Tht binds to these structures and its fluorescence increases; if, instead, the studied ligands and/or complexes are able to inhibit the aggregation, the Tht fluorescence increase is lower than that of the reference (Tht+P).

**Figure 9:**
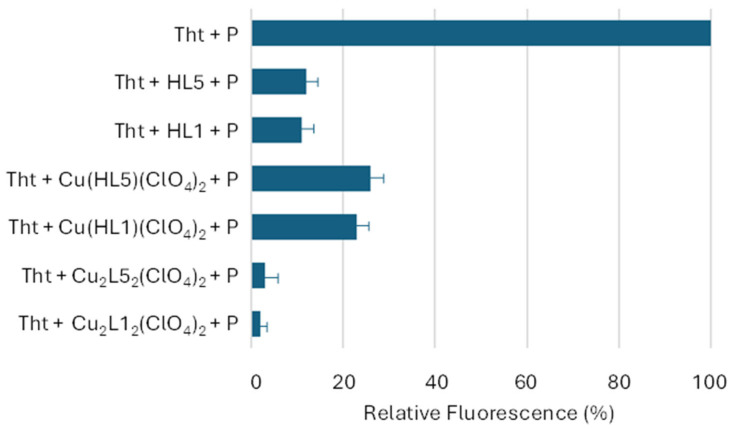
The fluorescence in the sample solutions present in the legend, where P = amyloid peptide. [ThT] = 10 μM; [P] = 10 μM; solutions of complexes, binders at concentration 10 μM, monitoring at 24 h.

The uncoordinated ligands significantly reduce aggregation (Figure 9). It is likely that the simple, flexible, and hydrophobic nature of HLx allows them to effectively interact with the hydrophobic regions of the peptides, which are those primarly involved in the aggregation. The test, repeated also in the presence of Cu(II) ions, created a strong interference during data acquisition; thus, the corresponding results could not be reported.

The synthesized mononuclear complexes showed reduced activity compared with the free ligands. As regards the dinuclear complexes (Figure 9), instead, they display the best antiaggregating performance.

## 3. Materials and Methods

### 3.1. Reagents

All chemicals and solvents were of analytical grade and obtained commercially from Merck (Darmstadt, Germany), used as received without additional purification. β-Amyloid (1–40) peptide was sourced from GenScript (Piscataway, NJ, USA). MilliQ-grade deionized water (Millipore, Burlington, MA, USA) was employed in the preparation of buffer solutions and for all spectroscopic analyses.

### 3.2. Synthesis

#### 3.2.1. Synthesis of the Mononuclear Complexes

The mononuclear complex Cu(HL1)(ClO_4_)_2_ was synthesized by mixing stoichiometric amounts of the ligand HL1 (yellow-colored solution) and Cu(ClO_4_)_2_ (pale blue-colored solution) in acetonitrile (CH_3_CN) or methanol (MeOH). Upon mixing, the resulting solution turned blue, indicating complex formation. For crystallization, both gradual solvent loss and gas–liquid diffusion approaches were utilized. The resulting blue crystals of Cu(HL1)(ClO_4_)_2_ were isolated and subsequently characterized.

Preparation of the mononuclear compound of HL5 was carried out using a procedure analogous to that used for the HL1-based system, employing either acetonitrile (CH_3_CN) or methanol (MeOH) as solvents.

Blue crystals of the complex were produced by means of controlled solvent evaporation and diffusion-based crystallization techniques. Crystallographic data have been deposited with the Cambridge Crystallographic Data Centre (CCDC) under reference numbers 2,448,897 (HL1 complex) and 2,448,898, 2,448,899 (HL5 complexes).

#### 3.2.2. Synthesis of the Dinuclear Complexes

Preparation of the dinuclear HL1 complex followed a modified method from the procedure described by Feringa and coworkers [55] for the ligand 2-{[di(2-pyridyl)methylamino]methyl}phenol. Initially, two equimolar methanolic solutions—one of the ligand HL1 (orange solution) and one of copper perchlorate (blue solution)—were mixed. Subsequently, a base (Et_3_N) was added to deprotonate the phenol group, promoting the formation of the dinuclear complex in which the negatively charged oxygen bridges the two metal ions.

The resulting mixture turned green, and a precipitate formed immediately. This solid was redissolved upon heating by the addition of CH_3_CN. After allowing the solution to return to ambient temperature, it was transferred to cold storage. After approximately one week, isolated crystals were obtained (33% yield). Powder XRD analysis was performed to assess the identity of the structure [56].

To prepare the HL5 Cu(II) dinuclear complex, an identical method to that outlined above was employed. The ligand and copper perchlorate were mixed in a methanolic solution, and the mixture was then treated with Et_3_N. A precipitate formed immediately, which was redissolved by the addition of CH_3_CN under heating. Following cooling, green crystals were obtained within approximately one week. The complex was isolated with a yield of 44%.

### 3.3. Instrumentation and Methodology

**UV–Vis** absorption spectra were acquired using Varian Cary 50, Cary 100, and Cary 5000 spectrophotometers, employing either standard 3 mL or reduced 1 mL volume quartz cuvettes, both with 1 cm of optical path. Ligand samples were prepared as 0.1 mM solutions in a 5% *v*/*v* MeOH/H_2_O mixture, whereas the complexes were dissolved in deionized water at concentrations ranging from 0.1 mM to 1.5 mM (the latter to better visualize d–d bands). Spectra for both ligands and their corresponding complexes were recorded under identical conditions.

**Potentiometric measurements** were performed using a Metrohm pH-meter system equipped with a semi-micro combination electrode, at 25 °C. The system was calibrated based on hydrogen ion concentration using HCl [57]. Ligands were prepared in a 5% (*v*/*v*) DMSO solution with water containing HCl/KCl with a constant ionic strength of I = 0.1 M to achieve a final concentration of 1 mM and 0.6 mM of HL1 and HL5, respectively. Titrations were conducted by incrementally adding 0.1 M KOH using a microsyringe at 25°C, spanning a pH interval from 2.5 to 11.5, under an argon atmosphere. The stability constants and complex stoichiometries were determined from the titration data using the SUPERQUAD and HYPERQUAD programs [58,59].

**Cyclic voltammetry** experiments were carried out using a BAS Cell C3 EC-epsilon potentiostat equipped with a conventional three-electrode system consisting of a glassy carbon working electrode (3 mm diameter, geometric surface 7 mm^2^), a platinum wire counter electrode (CE), and a silver/silver chloride reference electrode (Ag/AgCl, 3 M KCl). Voltammograms were recorded in phosphate-buffered saline (PBS; 10 mM, 150 mM NaCl, pH 7.8) containing 0.25 mM of the complex. The measurements were performed within a potential window from −600 mV to +400 mV, employing various scan rates (50 mV/s, 100 mV/s, 200 mV/s, 400 mV/s, and 800 mV/s).

**Catalytic measurement** assays were conducted using a Varian Cary 100 spectrophotometer with a 3 mL quartz cuvette (1 cm path length) for evaluating SOD-like activity. For the assessment of peroxidase-mimicking activity, a Varian Cary 50 instrument and identical cuvettes were utilized. To monitor the CAT-like activity, a 25 mL vial, equipped with a septum for solution injection, was used as a reactor and connected to a pressure transducer.

**The molecular structures of the complexes** were generated using Avogadro, an open-source software for molecular modeling and visualization (Version 1.2.0) [60].

## 4. Conclusions

This study aimed to synthesize and comprehensively characterize both mononuclear and dinuclear copper(II) complexes featuring an N_3_O-donor ligand framework, with analysis conducted in both solution and solid states. The primary goal was to evaluate their dual functionality—superoxide dismutase (SOD)-like and catalase-like reactivity—under physiological pH. A broader objective was to explore the potential application of these complexes in a novel multivalent therapeutic strategy for Alzheimer’s disease. To determine their diverse capabilities, a series of experimental approaches was employed (i) UV–Vis-monitored titrations of the ligands with Cu(II) ions to determine the stoichiometry and binding constants of in situ-formed complexes; (ii) pH-dependent UV–Vis spectroscopic titrations, to investigate the speciation and structural changes in solution; (iii) potentiometric titrations of ligands and complexes to derive protonation constants and pH-dependent species distribution profiles. In the cases of HL1 and HL5, the formation of mononuclear species [Cu(HLx)]^2+^ was confirmed, with higher stability for HL5 complexes; (iv) redox properties of the Cu(II)/Cu(I) couple were examined using cyclic voltammetry. The electrochemical profiles of Cu(HL1)(ClO_4_)_2_ and Cu(HL5)(ClO_4_)_2_ displayed quasi-reversible behavior, with the redox potential of the mononuclear complexes being better aligned with the electrochemical window required for superoxide radical dismutation; (v) SOD-like activity was assessed using the cytochrome c (CytC) indirect assay, wherein the rate of CytC reduction to ferricytochrome was monitored by UV–Vis spectroscopy. Among the studied systems, the HL5 complexes—particularly the mononuclear variant—demonstrated superior SOD-mimetic activity; (vi) catalase-like behavior was analyzed by quantifying oxygen evolution from hydrogen peroxide decomposition, with additional monitoring via UV–Vis spectroscopy to assess complex stability. These experiments revealed significant catalytic activity, as well as a general low stability of the complexes, likely due to radical-mediated degradation. (vii) Peroxidase-like activity was studied spectrophotometrically by tracking temporal changes in absorbance linked to substrate consumption (morin assay) or product formation (OPD assay). The results indicated that pro-oxidant properties are observed for all tested complexes. The latter is much higher for mononuclear complexes, while for the dinuclear complexes (especially HL5), it is less critical; (viii) an aggregation test, in the presence of 1–40 Aβ amyloids, was carried out by monitoring the fluorescence of ThT over a period of about 24 h. From this test, it emerged that all compounds affect the aggregation, with the dinuclear compounds (especially with HL1) being the most effective.

The results obtained highlight that all complexes studied show the desired activities. On one hand, the ligands function as chelators, and their mononuclear complexes offer better accessibility to the active site for O_2_^−^• dismutation, albeit with a more pronounced residual oxidative activity. On the other hand, the dinuclear species prove more advantageous, exhibiting high efficiency in H_2_O_2_ dismutation and Aβ anti-aggregation while avoiding strong oxidative behavior—underscoring the structural and mechanistic benefits of coordinating two copper ions. Future experiments will be useful to fine-tune the redox properties of copper sites and to improve the stability of the complexes under oxidative conditions.

## Figures and Tables

**Table 1:** The overall (log*β_i_*) and stepwise (log*K_i_*) protonation constants of the ligands HL1 and HL5, obtained from potentiometric titration.

Equilibrium Reaction	log*β_i_* HL1	log*β_i_* HL5	Equilibrium Reaction	log*K_i_* HL1	log*K_i_* HL5
Lx^−^ + H^+^ 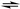 HLx	9.99 ± 0.01	9.52 ± 0.01	Lx^−^ + H^+^ 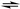 HLx	9.99	9.52
Lx^−^ + 2H^+^ 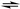 [(HLx)H]^+^	16.29 ± 0.02	15.77 ± 0.02	HLx + H^+^ 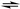 [(HLx)H]^+^	6.30	6.25
Lx^−^ + 3H^+^ 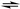 [(HLx)H_2_]^2+^	21.53 ± 0.02	20.71 ± 0.02	[(HLx)H]^+^ +H^+^ 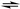 [(HLx)H_2_]^2+^	5.24	4.94
Lx^−^ + 4H^+^ 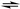 [(HLx)H_3_]^3+^	25.24 ± 0.02	24.43 ± 0.02	[(HLx)H_2_]^2+^ +H^+^ 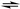 [(HLx)H_3_]^3+^	3.71	3.72

**Table 2:** Overall (log*βi*) and stepwise (p*Ki*) stability constants of in situ-formed complexes with HL1 (x = 1) and HL5 (x = 5), obtained from potentiometric titration.

Species	log*β_i_* x = 1	log*β_i_* x = 5	p*K_i_* x = 1	p*K_i_* x = 5
[Cu(HLx)H]^3+^	22.67 ± 0.06	23.43 ± 0.08		
[Cu(HLx)]^2+^	16.84 ± 0.08	18.04 ± 0.10	5.83	5.39
[Cu(Lx)]^+^	9.73 ± 0.08	11.51 ± 0.10	7.11	6.53
[Cu(Lx)(OH)]	0.03 ± 0.09	1.77 ± 0.11	9.70	9.74
[Cu(Lx)(OH)_2_]^−^	−10.42 ± 0.09	−8.56 ± 0.11	10.45	10.33

**Table 3:** E_pc_, E_pa_, E_1/2_, ΔE, │i_a_/i_c_│ values obtained from the cyclic voltammograms of the complexes. The potentials are referred to NHE (V_NHE_ = V_Ag/AgCl_ + 0.210 V).

Complexes	E_pc_ (V)	E_pa_ (V)	E_1/2_ (V)	ΔE (V)	│i_a_/i_c_│
Cu(HL1)(ClO_4_)_2_	−0.177	−0.092	−0.135	−0.085	0.61
Cu + HL1 (in situ)	−0.223	−0.143	−0.183	−0.080	1.35
Cu(HL5)(ClO_4_)_2_	−0.202	−0.096	−0.149	−0.106	0.52
Cu + HL5 (in situ)	−0.216	−0.134	−0.175	−0.082	1.44
Cu_2_L1_2_(ClO_4_)_2_	−0.229	−0.150	−0.190	−0.079	1.04
Cu_2_L5_2_(ClO_4_)_2_	−0.219	−0.138	−0.179	−0.081	0.90

**Table 4:** The summary of SOD activity expressed as IC_50_ and log (*k_cat_*) for the native Cu-ZnSOD and mononuclear and dinuclear Cu(II) complexes; the values were obtained with (a) NBT assay or (b) CytC assay; (c) dib = [2-(4,5-dihydro-1H-imidazol-2-yl)-1Hbenzimidazole-N,N,O], (d) HBPMPA = N-propanoate-N,N-bis-(2-pyridylmethyl)amine, (e) TAAP, that is obtained by self-condensation of 5-amino-3-methyl-l- phenylpyrazole-4-carbaldehyde (AMsPC) in the presence of copper(II), (f) tppen, that is derived from tris(2-pyridilmethyl)amine, (g) 2-({[di(2-pyridyl)methyl]amino}methyl)-4-nitrophenol. (h) Condition adopted: [CytC] = 10 μM, [catalase] = 15 μg/mL, [xanthine] = 40 μM, [xanthine oxidase] = 0.0053 U/mL, phosphate buffer 50 mM, pH = 7.8.

Complexes	IC_50_ (μM)	Log (k_cat_)	Ref.
Native Cu-ZnSOD	0.04 ^a^	9.30	[40,41]
CuSO_4_	72.2 ^b^	~6	[42]
Cu(HL1)(ClO_4_)_2_	1.41 ^b^	6.27 ^h^	This work
Cu_2_(L1)_2_(ClO_4_)_2_	1.99 ^b^	6.13 ^h^	This work
Cu(HL5)(ClO_4_)_2_	1.04 ^b^	6.41 ^h^	This work
Cu_2_(L5)_2_(ClO_4_)_2_	1.28 ^b^	6.31 ^h^	This work
Cu(dib)Cl_2_^c^	0.09 ^a^	-	[43]
[Cu(BPMPA)]^+ d^	1.04 ^b^	7.09	[44]
Cu(TAAP)(NO_3_)_2_^e^	0.55 ^a^	7.26	[8]
Cu_2_(tppen)Cl_4_^f^	0.54 ^b^	-	[45]
[Cu_2_(L-RNH_2_)(H_2_O)_2_](ClO_4_)_3_	0.66	6.59	[28]
[CuZn(salpn)Cl_2_	3.2 ^a^	-	[46]
[Cu(py_2_bn)]^2+^	0.078 ^a^	-	[38]
Cu_2_L^3^_2_^g^	0.072 ^b^	7.55	[47]

**Table 5:** Average values, obtained from triplicate experiments, of R_max_ and of O_2_ µmol generated for the complexes. Conditions: (a) [catalyst] = 200 μM, [H_2_O_2_] = 20–30 mM in BBS 50 mM, pH = 7.8, (b) HL = 2-({[di(2-pyridyl)methyl]amino}methyl)phenol) derivatives, [Catalyst] = 100 μM, [H_2_O_2_] = 30 mM, BBS 50 mM, pH = 7.8), (c) L = 2,6-bis[(bis(2-pyridylmethyl)amino)methyl]-phenol derivatives [Catalyst] = 100 μM, [H_2_O_2_] = 22.5 mM, BBS 50 mM, pH = 7.8), (d) CATm1 = peptide sequence PHYKHRLH [Catalyst] = 100 μM, [H_2_O_2_] = 30 mM, MOPS buffer (50 mM, pH 7.5), (e) values are within ±10% error.

Complexes	Induction Time (min)	R_max_ (μM/min)	Time (min)	O_2_ (mmol/L)	Ref.
Cu(HL1)(ClO_4_)_2_^(a)^	250	0.25	950	7.6	This work ^(e)^
Cu(HL5)(ClO_4_)_2_^(a)^	-	0.80	1300	9.8	This work ^(e)^
Cu_2_(L1)_2_(ClO_4_)_2_^(a)^	200	0.20	1300	9.2	This work ^(e)^
Cu_2_(L5)_2_(ClO_4_)_2_^(a)^	-	0.36	950	7.7	This work ^(e)^
Cu_2_L^1^_2_^(b)^	10	52	240	5.0	[47]
Cu_2_L^2^_2_^(b)^	-	264	240	5.4	[47]
Cu_2_L^3^_2_^(b)^	-	258	240	5.4	[47]
[Cu_2_(L-CH_3_)](ClO_4_)_3_^(c)^	-	45	180	6.5	[28]
[Cu_2_(L-RNH_2_)](ClO_4_)_3_^(c)^	-	55	180	6.9	[28]
[Cu_2_(L-RCOOH)](ClO_4_)_3_^(c)^	30	44	180	7.9	[28]
(CATm1)Cu_2_^(d)^	-	273	3	0.3	[50,51]

## Data Availability

The data are available on request from the corresponding authors.

## Supplementary Materials

The following supporting information can be downloaded at: https://www.mdpi.com/article/10.3390/molecules30163419/s1, Figures S1–S23: Characterization of the ligands; Figures S24–S37: Characterization of the complexes; Figures S38–S42: Titration of the ligands with Cu(II) and pH-dependent behavior of the complexes; Figure S43: Cyclic voltammetry; Figure S44: H_2_O_2_ dismutation in the presence of KBr; Figures S45–S48: Stability monitoring by UV–Vis; Figures S49–S53: Absorbance variation during morin oxidation; Figures S54–S58: Absorbance variation during OPD oxidation. Table S1: Crystallographic details for [Cu(HL1)(CH_3_CN)ClO_4_](ClO_4_)·CH_3_CN (1), [Cu(HL5)(CH_3_CN)ClO_4_](ClO_4_) (2) and [Cu(HL5)(MeOH)ClO_4_](ClO_4_) (3); Table S2: Selected bond lengths (Å) and angles (°) for [Cu(HL1)(CH_3_CN)ClO_4_](ClO_4_)·CH_3_CN (1); Table S3: Selected bond lengths (Å) and angles (°) for [Cu(HL5)(CH_3_CN)ClO_4_](ClO_4_) (2) and [Cu(HL5)(MeOH)ClO_4_](ClO_4_) (3). Refs. [56,61,62,63,64,65,66] are cited in Supplementary Materials.
