# Supplementary material for: The Chelating Abilities of Tertiary Amines with N-O-Donors Towards Cu(II) Ions and the Catalytic Properties of the Resulting Complexes
Source: Molecules. 2025 Aug 19;30(16):3419. doi: 10.3390/molecules30163419 (PMC12388300; doi:10.3390/molecules30163419)
Supplement: Supplementary file 1 [file molecules-30-03419-s001.zip › molecules-3667914-supplementary.pdf]

Supporting Information for

The chelating abilities of tertiary amines with N-O-donors towards Cu(II) ions and the catalytic properties of the resulting complexes

Martina Zonzin, Martina Chianese, Andrea Squarcina, Degnet Melese Dereje, Ambra Campofelice, Alessia Da Fermo, Federica Belluti, Nadia Marino, Filip Dębicki, Aleksandra Kotynia, Aleksandra Marciniak, Justyna Brasuń, Mauro Carraro

1. Reagents
2. Instrumentation
3. X-ray data collection and structure refinement
4. Synthesis and characterization of the ligands
5. Synthesis and characterization of the complexes
6. Chelating ability
7. Cyclic voltammetry
8. Catalytic activity

1. Reagents

Commercially available reagents and solvents were provided by Merck and used with no further purifications. β-Amyloid (1-40) was purchased from GenScript. MilliQ-deionized water (Millipore) was used for preparing the buffer solutions and for spectroscopic measurements.

2. Instrumentation

UV-Vis spectra were recorded with spectrometers Varian Cary 50, Cary 100 or Cary 5000, using standard (3 mL) or reduced (1 mL) volume quartz cuvettes with 1 cm of optical path.

FT-IR spectra were measured, preparing KBr pellets, with a Nicolet 5700-Thermo Electron Corporation instrument (Hemel Hempstead, UK). Throughout the vibrational bands assignment, the following symbols were used: w: weak signal, m: medium signal, s: strong signal, b: broad signal.

^{1}H- and ^{13}C **NMR** spectra were recorded on a Bruker spectrometer working at 600 and 150 MHz, respectively. The chemical shifts were assigned using $Si(CH_3)_4$ as reference (δ ^{1}H-NMR = 0 ppm). Multiplicity is described as s: singlet, d: doublet, t: triplet, td: triplet of doublets, m: multiplet.

ESI-MS spectra were collected using an Agilent Technologies MSD SL Trap spectrometer with ESI source. Samples were dissolved in CH_3CN, CH_3CN/H^+ (0.1% v/v HCOOH), MeOH, or $MeOH/H^+$ (0.1% v/v HCOOH) or H_2O, and injected into the ion source at a flow rate of 0.05 mL/min.

CHN elemental analyses were performed in duplicate by a Thermo Fisher Flash 2000 instrument.

UHPLC-MS analyses were run on a Waters ACQUITY ARC UHPLC/MS system consisting of a QDA mass spectrometer equipped with an electrospray ionization interface and a 2489 UV/ Vis detector; the detected wavelength was 365 nm.

Powder X-ray Diffraction (PXRD). PXRD measurements were carried out with a Bruker D8 Advance diffractometer using Cu Kα radiation (λ = 1.5406 Å) at a voltage of 40 kV and current of 40 mA. PXRD patterns were collected for 2θ = 8–80°, with a step size of 0.03°. All measurements were conducted at room temperature (298 K) in continuous scan mode.

3. **X-ray data collection and structure refinement**

Single-crystals of the mononuclear complexes [Cu(HL1)(CH_3CN)ClO_4](ClO_4)·CH_3CN (**1**), [Cu(HL5)(CH_3CN)ClO_4](ClO_4) (**2**) and [Cu(HL5)(MeOH)ClO_4](ClO_4) (**3**), suitable for the diffraction analysis, were carefully chosen under the microscope, mounted on a nylon loop with the aid of either Paratone oil or Loctite glue, positioned over a goniometer head and transferred in the diffractometer collection chamber. **1** and **2** were collected at room temperature, while **3** was collected at 153 K. The measurements were carried out with an Oxford Diffraction Gemini E diffractometer, equipped with a 2 K × 2 K EOS CCD area detector and sealed-tube Enhance (Mo) and (Cu) X-ray sources. Data collection, reduction and finalisation were carried out through the CrysAlisPro software. The structures were solved by standard direct methods and subsequently completed by Fourier recycling by using the SHELXTL software packages. The obtained models were refined with the 2019/3 version of SHELXL against F^2 on all data by full-matrix least squares,[1] using established methods.[2] Both the coordinated and uncoordinated perchlorate anions in **2** and **3** show positional disorder of the oxygen atoms only; the hydrogen atom of the coordinated methanol molecule in **3** was unstable during the refinement and thus it has been excluded from the final model. A summary of the crystallographic data and refinement details for **1**-**3** is given in Table S1, while selected bond distances and angles are given in Tables S2 (**1**) and S3 (**2**-**3**). Crystallographic data have been deposited at the Cambridge Crystallographic Data Centre and have been assigned CCDC reference numbers 2448897 (**1**), 2448898 (**2**) and 2448899 (**3**).

[1] Sheldrick, G. M. *Acta Crystallogr.* **2015**, 3-8.

[2] (a) Müller, P. *Crystallogr. Rev.,* **2009**, 15, 57; (b) Müller, P.; Herbst-Irmer, R.; Spek, A. L.; Schneider, T. R.; Sawaya, M. R. Crystal Structure Refinement: A Crystallographer's Guide to SHELXL, ed. Müller, P.; IUCR Texts on Crystallography, Oxford University Press, Oxford, 2006.

Table S1. Crystallographic details for $[Cu(HL1)(CH_3CN)ClO_4](ClO_4)\cdot CH_3CN$ (**1**), $[Cu(HL5)(CH_3CN)ClO_4](ClO_4)$ (**2**) and $[Cu(HL5)(MeOH)ClO_4](ClO_4)$ (**3**).

	**1**	**2**	**3**
Ligand, solvent	**HL1**, CH_3CN	**HL5**, CH_3CN	**HL5**, MeOH
Empirical formula	$C_{23}H_{25}Cl_2CuN_5O_9$	$C_{22}H_{24}Cl_2CuN_4O_{10}$	$C_{21}H_{24}Cl_2CuN_3O_{11}$
Formula weight	649.92	638.89	628.87
T/K	298(2)	294(2)	153(2)
Wavelength/Å	1.54184	0.71073	0.71073
Crystal system	Monoclinic	Monoclinic	Monoclinic
Space group	*Cc*	$P2_1/c$	$P2_1/n$
a/Å	10.5363(11)	13.4520(9)	9.4686(4)
b/Å	33.429(4)	18.1046(10)	33.7302(17)
c/Å	8.3999(8)	12.1262(9)	8.3418(4)
β/°	107.498(12)	113.810(9)	100.840(4)
V/Å^3	2821.7(5)	2701.9(3)	2616.6(2)
Z	4	4	4
D_{calc}/gcm^{-3}	1.530	1.571	1.596
μ/mm^{-1}	3.351	1.067	1.102
F(000)	1332	1308	1288
ϑ range for data collection	2.644 – 50.431°	2.218 – 29.358	2.501 – 26.815
Refl. coll.	4115	48520	20310
Refl. indep. (R_{int})	2089 (0.0319)	6835 (0.0697)	4774 (0.0836)
Data/restraints/parameters	2089/341/364	5937/550/448	4774 / 252 / 399
Goodness-of-fit[c] on F^2	1.170	1.036	1.098
R_1[a] [I>2σ(I)] (all)	0.0526 (0.0640)	0.0519 (0.1212)	0.0617 (0.0787)
wR_2[b] [I>2σ(I)] (all)	0.1410 (0.1457)	0.1176 (0.1493)	0.1328 (0.1471)
Abs. struct. par.	-0.01(5)	n/a	n/a
Ext. coef.	0.00016(9)	n/a	n/a
Largest diff. peak and hole/e·Å-3	0.364 and -0.369	0.552 and -0.374	1.072 and -0.568

[a] $R_1 = \sum(|F_o| - |F_c|)/\sum|F_o|$. [b] $wR_2 = [\sum w(F_o^2 - F_c^2)^2/\sum w(F_o^2)^2]^{1/2}$. [c] $S = [\sum w(|F_o| - |F_c|)^2/(N_o - N_p)]^{1/2}$.

4. **Synthesis and characterization of the ligands**

BPMA (bis-(2-pyridylmethyl) amine), a common building block for both ligands, was synthesized via reductive amination of pyridine-2-carboxaldehyde and 2-(aminomethyl) pyridine, following a procedure reported by *Hamman et al.*[3] reaction between the two compounds gives the intermediate imine, which is reduced in the second step by $NaBH_4$ to obtain a brown oil (*Figure S1*).

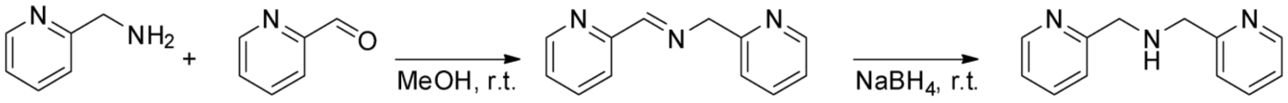

Figure S1: *Synthesis of* ***BPMA***.

A solution of pyridine-2-carboxaldehyde (3.02 g, 28.2 mmol) in 10 mL of methanol, pre-cooled to 0 °C, was added dropwise to a stirred solution of 2-(aminomethyl)pyridine (3.02 g, 27.9 mmol) in 10 mL of methanol. The resulting dark yellow solution was stirred at room temperature for 1 hour. Subsequently, $NaBH_4$ (0.391 g, 10.3 mmol) was added in portions at 0 °C, leading to the formation of a yellow foam and a colour change to dark orange. After stirring at room temperature for 12 hours, the reaction mixture was poured onto ice and acidified to pH 4 using 2 M HCl, yielding a dark orange solution. The mixture was concentrated under reduced pressure to remove the solvent, to obtain a dark oil, which was then dissolved in 14 mL of water. The resulting brown aqueous solution was extracted with CH_2Cl_2 (5 × 10 mL) until the organic layer became colourless. The aqueous layer was then basified to pH 10 with saturated Na_2CO_3 and extracted with CH_2Cl_2 (8 × 10 mL). The combined organic extracts were dried over anhydrous Na_2SO_4, filtered, and concentrated under vacuum to afford a dark brown oil (19.6 mmol, 70% yield). ^1H NMR (600 MHz, $CDCl_3$): δ 8.55 (dd, *J* = 4.8, 1.2 Hz, 2H. H-3, H3′), 7.64 (td, *J* = 7.6, 1.8 Hz, 2H, H-5,H-5′), 7.35 (dd, *J* = 7.8, 1.8 Hz, 2H, H-6,H-6′), 7.15 (ddd, *J* = 7.5, 4.8, 1.2 Hz, 2H, H-4,H-4′), 3.98 (s, 4H, CH_2 x 2), 2.40 (br, 1H, NH), (*Figure S2*). ^{13}C NMR (151 MHz, $CDCl_3$): δ 159.66 (C1, C1′), 149.46 (C3, C3′), 136.62 (C5, C5′), 122.44 (C6, C6′), 122.12 (C4, C4′), 54.84 (CH_2 x 2), (*Figure S3*). ESI-MS (*m*/*z*) Calcd for: $C_{12}H_{13}N_3$ 199.25, $[HBPMA]^+$ (*m*/*z* calc: 200.12), found 200.1 [M+1], (*Figure S6*).

[3] Hamman, J.N.; Rolff, M.; Tuczek, F., *Dalton Trans.*, **2015**, 44, 3251-3258.

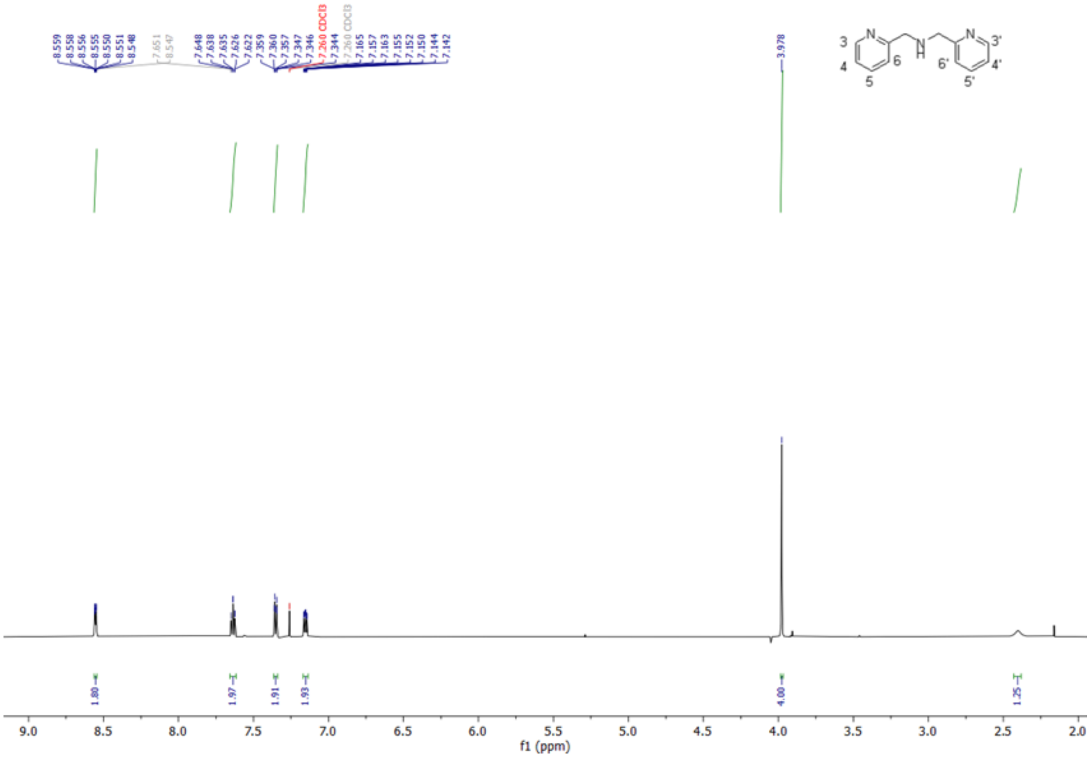

Figure S2.^{1}*H NMR (CDCl$_3$, 600 MHz) spectrum of compound BPMA*.

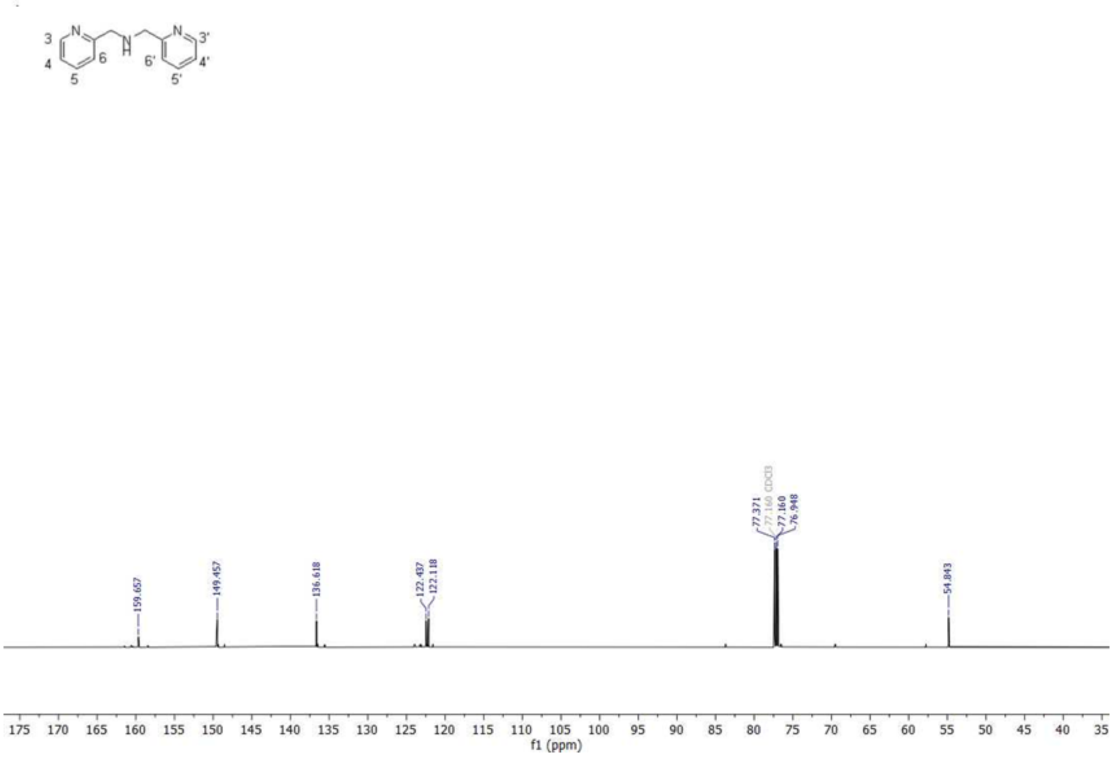

Figure S3.^{13}*C NMR (CDCl$_3$, 150 MHz) spectrum of compound BPMA.*

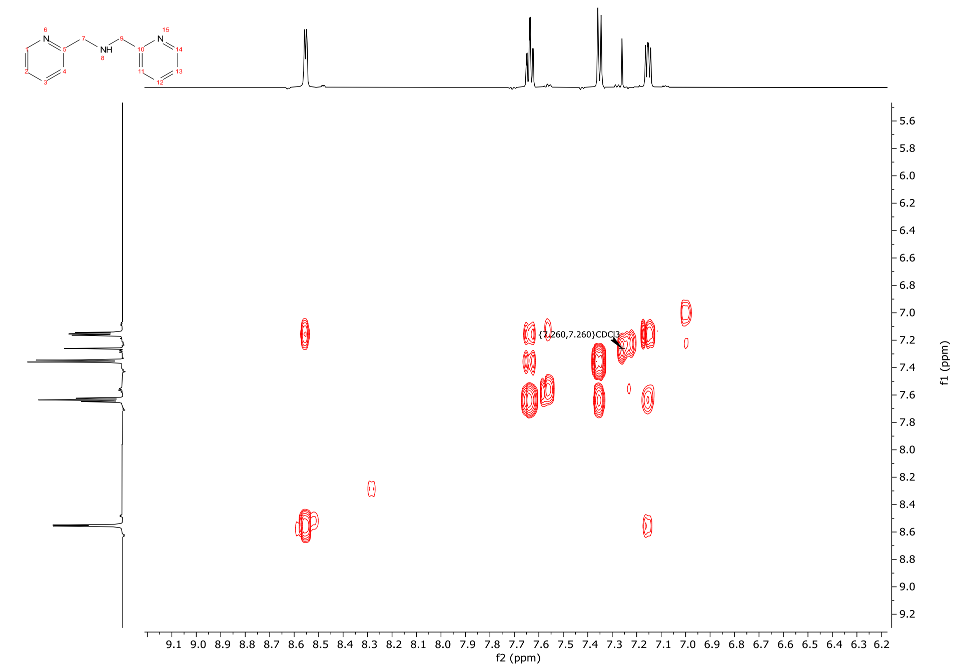

Figure S4. *^1H–^1H COSY spectrum of compound BPMA.*

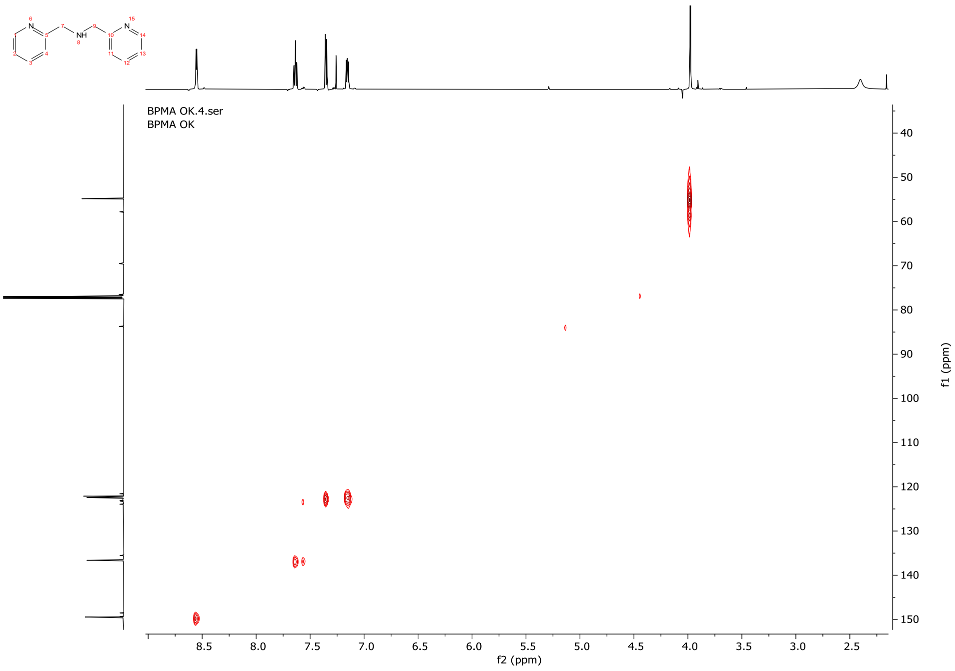

Figure S5. *^1H–^{13}C HSQC spectrum of compound BPMA.*

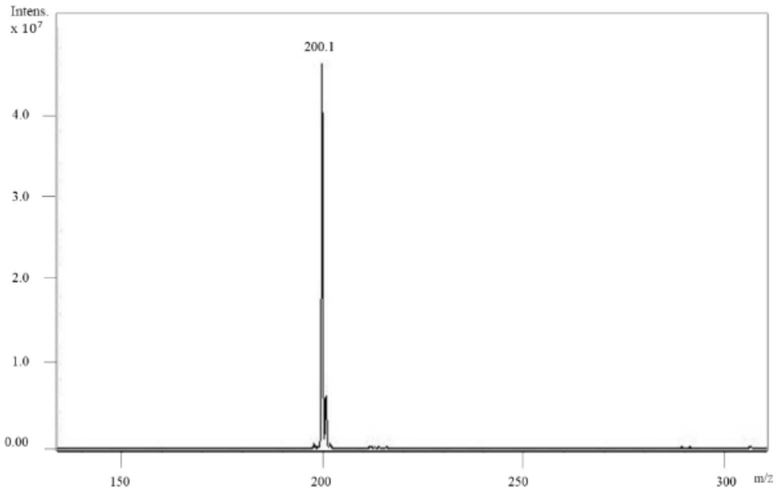

Figure S6: *ESI-MS (+) spectrum (CH_3CN, m/z) of BPMA.*

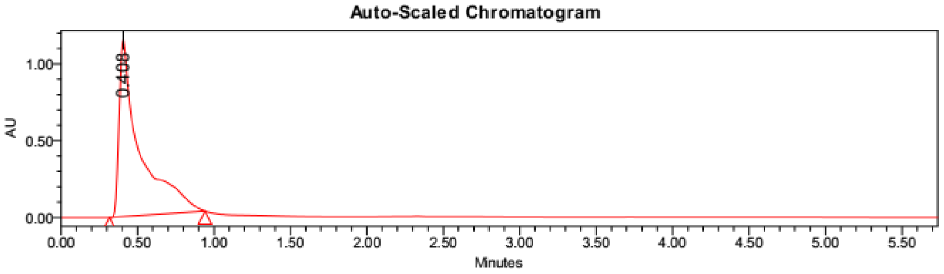

Peak Results

	Name	RT	Area	Height	% Area
1		0.408	10893613	1150580	100.00

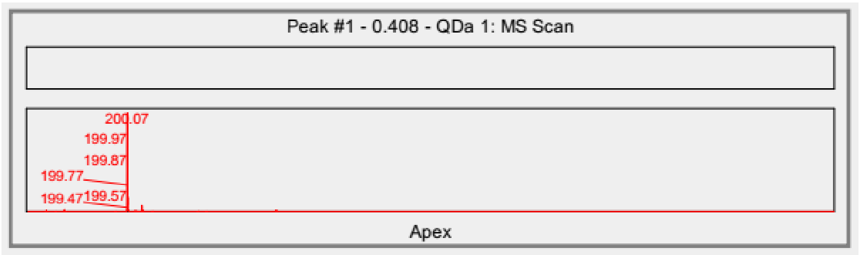

Figure S7. *UHPLC-MS chromatogram of compound BPMA.*

Synthesis of HL1

The ligand N-(2-hydroxylbenzyl)-N,N-bis(2-pyridylmethyl)amine (HL1) was synthesized via reductive amination following *Connor et al.*[4] reaction between BPMA and salicylaldehyde yield an imine, which then is reduced to a tertiary amine by $NaBH_3CN$ (*Figure S8*).

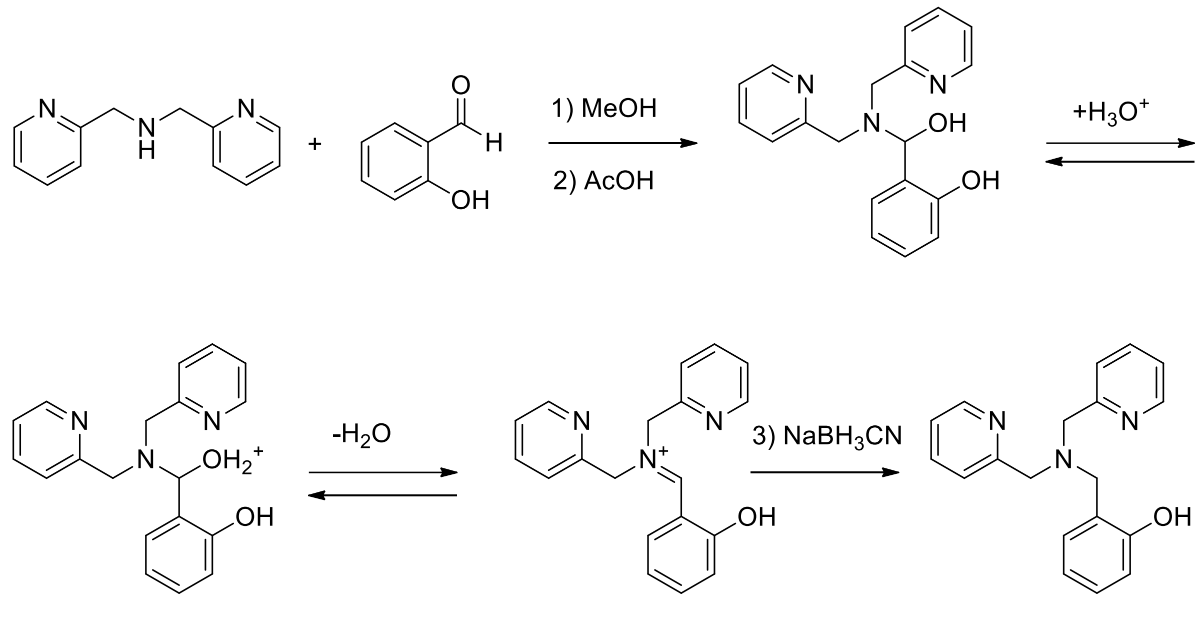

Figure S8: *Synthesis of* ***HL1****.*

Salicylaldehyde (0.710g, 5.82 mmol) was dissolved in 17 mL of MeOH and combined with BPMA (1.1490 g, 5.77 mmol) in 13 mL of MeOH. The solution appeared dark yellow. Three drops of glacial acetic acid were added, followed by the dropwise addition of $NaBH_3CN$ (0.195 g, 9.10 mmol in 2.6 mL MeOH). The resulting solution was refluxed for 1 h at 60°C and stirred overnight at room temperature. Upon addition of 2M HCl, to adjust the pH to 4.1, the colour changed from orange to light red. The solution was dried in vacuo, dissolved in 12.5 mL of saturated Na_2CO_3 and then extracted with $CHCl_3$ (3 x 30 mL). The organic phase was dehydrated with Na_2SO_4, filtered, and evaporated to obtain a dark red oil. The oil was purified with a chromatographic column (silica, DCM/MeOH 8:2, with NH_4Cl-saturated eluent, to improve the elution of the amine, which otherwise interacts too strongly with silica, slowing down the run). After the purification, the product was dissolved in dichloromethane and extracted with water (4 x 10 mL), to remove the NH_4Cl. The organic phase was dried with Na_2SO_4 and filtered. The solvent was removed under vacuum to provide a red oil with 54% yield.

^{1}H NMR (600 MHz, $CDCl_3$): δ 8.55 (dd, *J* = 4.8, 1.2 Hz, 2H. H-3, H3′), 7.61 (td, *J* = 7.6, 1.8 Hz, 2H, H-5,H-5′), 7.36 (d, *J* = 7.8, 2H, H-6,H-6′), 7.19-7.15 (m, 3H, H-4, H-4′, Ar″), 7.06 (dd, *J* = 7.4, 1.8 Hz, 1H, Ar″), 6.91 (dd, *J* = 8.1, 1.2 Hz, 1H, Ar″), 6.77 (td, *J* = 7.4, 1.2 Hz, 1H, Ar″), 3.86 (s, 4H, CH_2 x2), 3.78 (s, 2H, CH_2), (*Figure S9*). ^{13}C NMR (151 MHz, $CDCl_3$) δ 158.11 (Cq), 157.41(Cq), 148.73 (C3, C3′), 136.74 (C5, C5′), 130.07 (C6″), 128.96 (C4″), 123.13 (C6, C6′), 122.68 (C2″), 122.15 (C4, C4′), 118.77 (C5″), 116.41 (C3″), 58.91(CH_2 x 2), 56.81(CH_2), (*Figure S10*). ESI-MS (*m*/*z*) Calcd for: $C_{19}H_{19}N_3O$ 305.4, $[H2L1]^+$ (*m/z* calc: 306.16), found 306.2 [M+1], (*Figure S13*). Elemental analysis: C, 74.73; H, 6.27; N, 13.76; *found:* C, 73.98; H, 6.59; N, 13.96).

4 Connor, G. P.; Mayer, K. J.; Tribble, C. S.; McNamara, W. R.; *Inorg. Chem.*, **2014**, 53, 5408−5410.

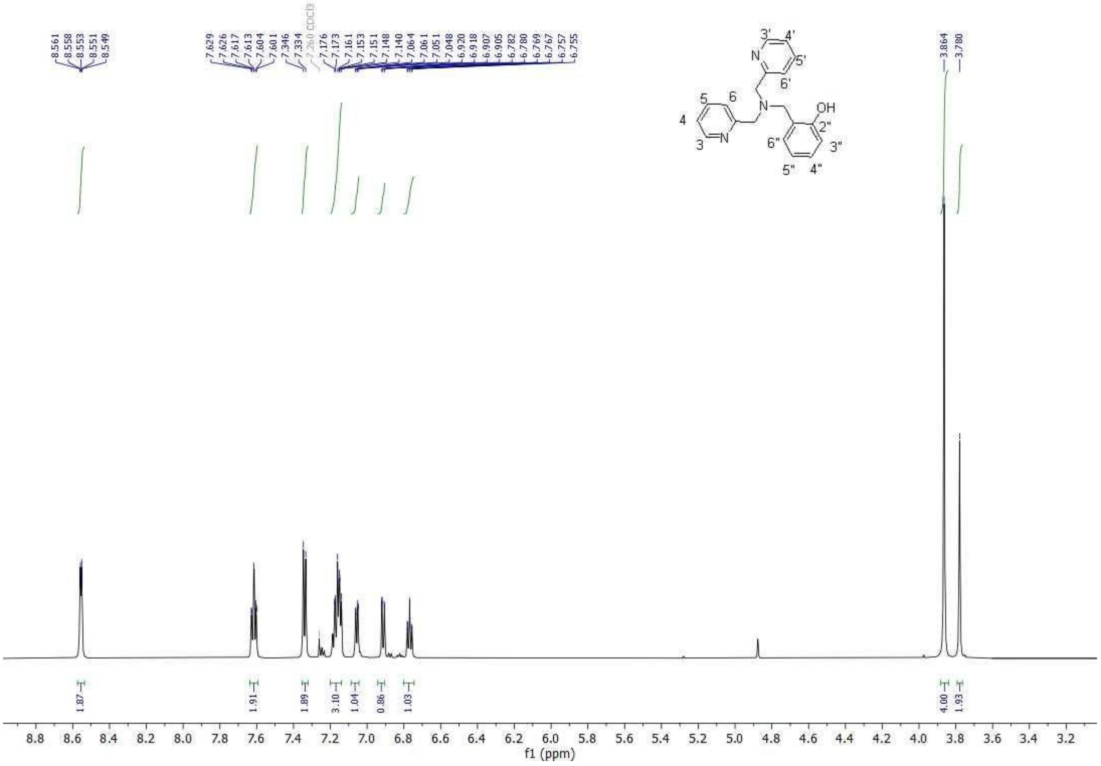

Figure S9. *^{1}H NMR (CDCl₃, 600 MHz) copy of compound* ***HL1***.

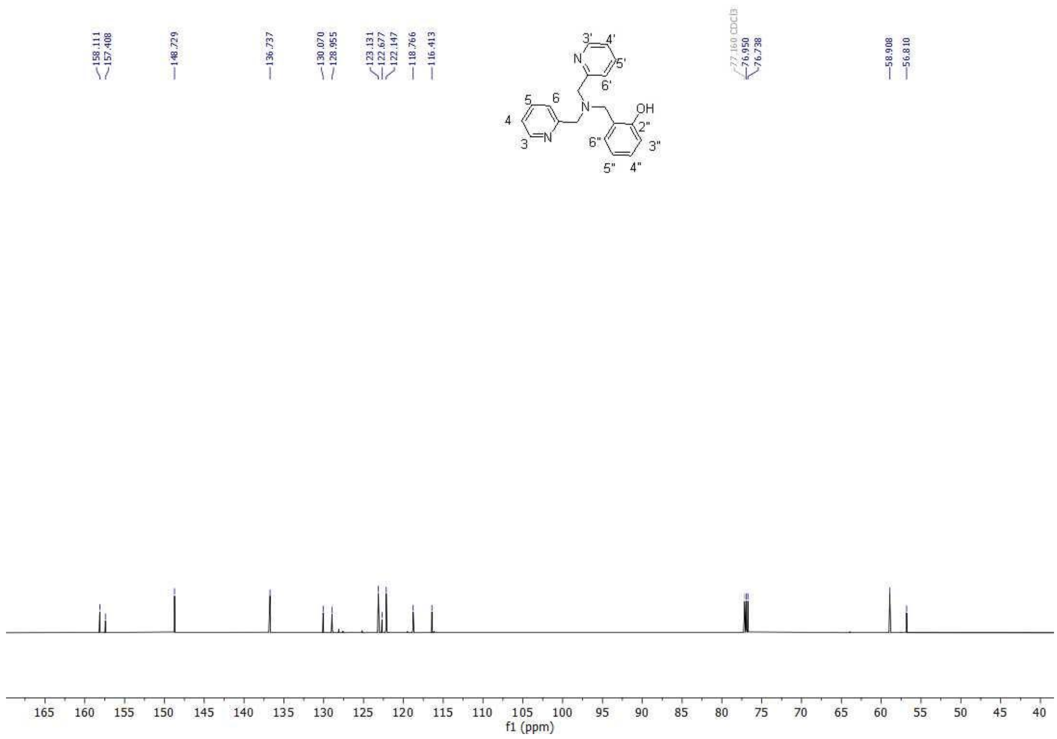

Figure S10. *^{13}C NMR (CDCl₃, 150 MHz) copy of compound* ***HL1***.

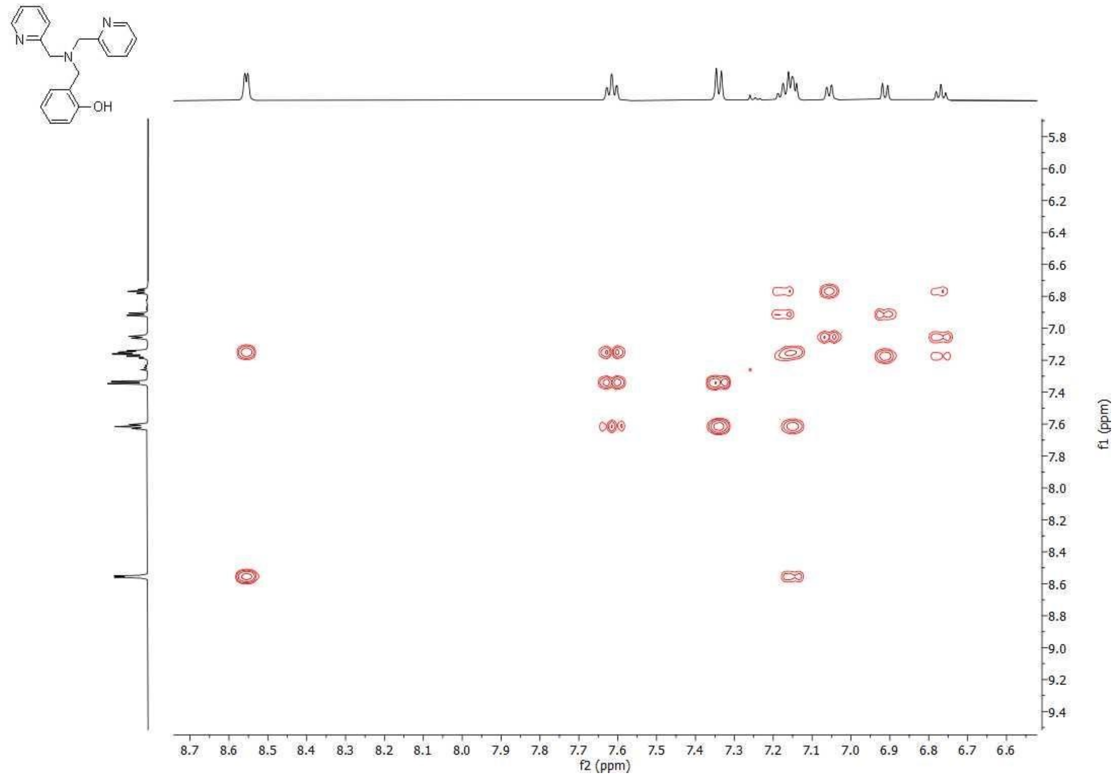

Figure S11.^{1}H–^{1}H *COSY copy of compound* ***HL1***.

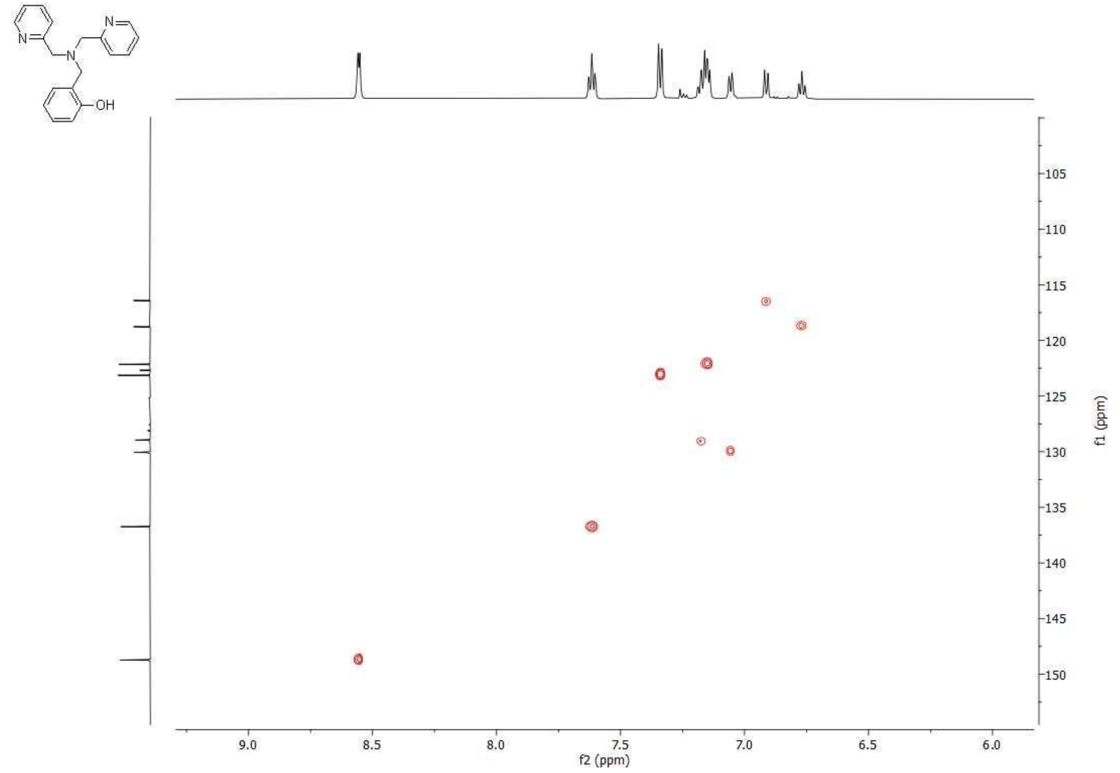

Figure S12.^{1}H–^{13}C *HSQC copy of compound* ***HL1***.

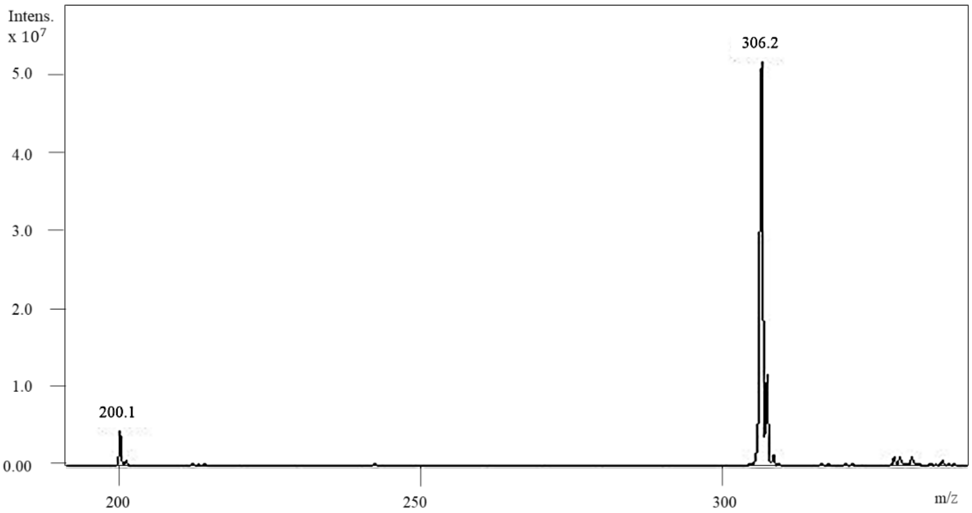

Figure S13.*ESI-MS (+)* spectrum *(CH3CN, m/z) of compound* ***HL1***.

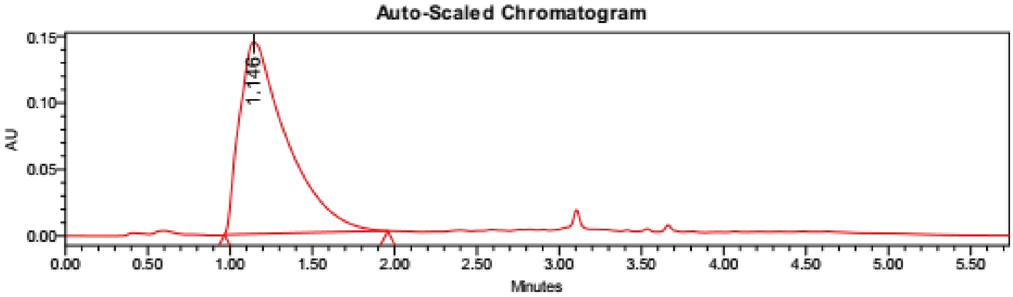

Peak Results

	Name	RT	Area	Height	% Area
1		1.146	2891677	144079	100.00

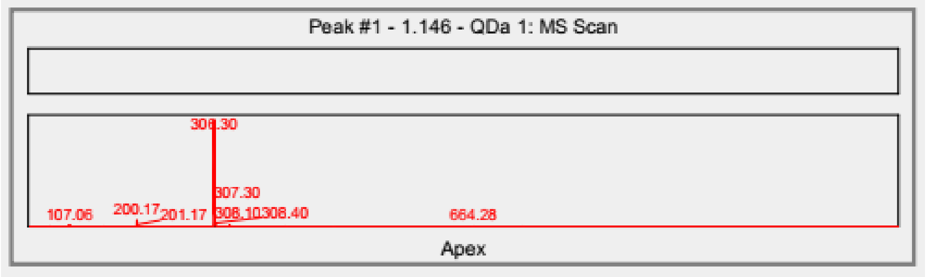

Figure S14.*UHPLC-MS chromatogram for compound* ***HL1***.

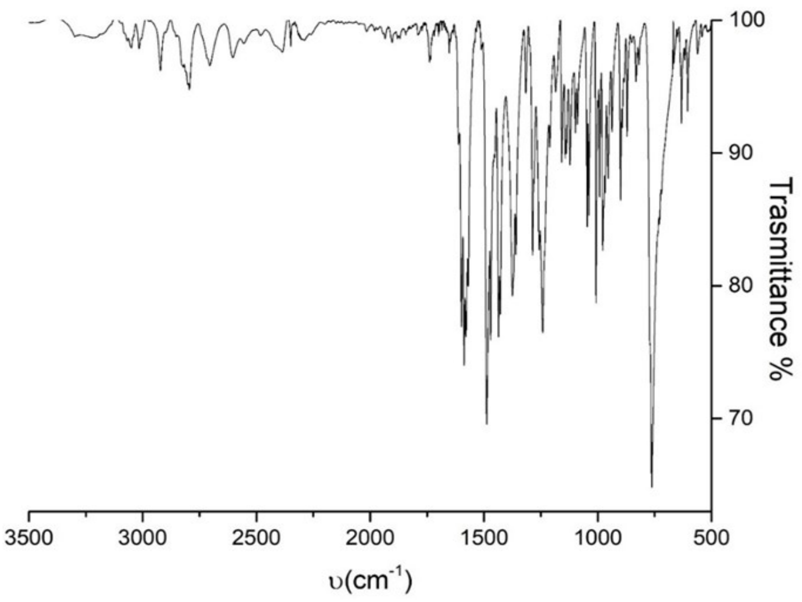

Figure S15: *FT-IR spectrum (KBr) of HL1.*

Synthesis of HL5

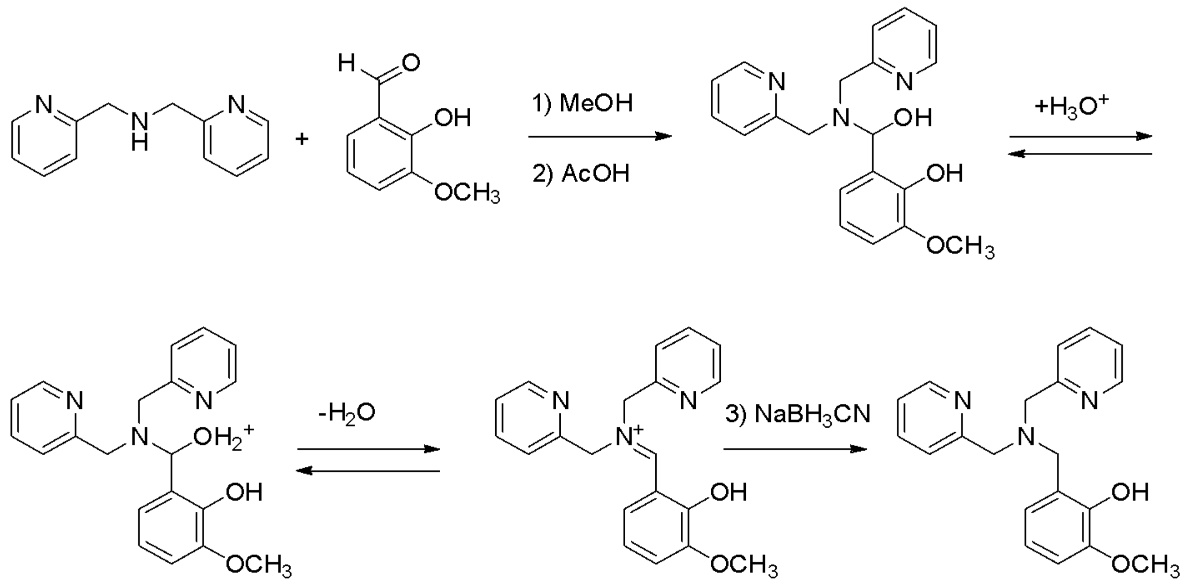

Figure S16: *Synthesis of HL5.*

2-{[Bis(pyridin-2-ylmethyl)amino]methyl}-6-methoxyphenol (HL5) was synthesized following the same procedure used for the ligand HL1, using o-vanillin as reactant (*Figure S16*). BPMA (0.886 g, 4.5 mmol), o-vanillin (0.658 g, 4.3 mmol) and $NaBH_3CN$ (0.197g, 3.13 mmol in 2.5 mL MeOH) were reacted in 25 mL of MeOH. The product was purified with a chromatographic column, using, as eluent, a DCM/MeOH 8:2 mixture saturated with NH_4Cl, obtaining a dark orange oil, with 44% yield.

^{1}H NMR (600 MHz, $CDCl_3$): δ 8.55 (dd, *J* = 4.8, 1.2 Hz, 2H. H-3, H3′), 7.61 (td, *J* = 7.6, 1.8 Hz, 2H, H-5,H-5′), 7.38 (d, *J* = 7.8 Hz, 2H, H-6,H-6′), 7.15 (ddd, *J* = 7.5, 4.8, 1.1 Hz, 2H,H-4, H-4′), 6.82 (dd, *J* = 7.8, 1.8 Hz, 1H, H6″), 6.73 (t, *J* = 7.8 Hz, 1H, H-5″) 6.71 (m, dd, *J* = 7.8, 1.8 Hz, 1H, H4″), 3.90 (s, 3H, OCH_3), 3.87 (s, 4H, CH_2 x 2), 3.80 (s, 2H, CH_2), (*Figure S17*). ^{13}C NMR (151 MHz, $CDCl_3$): δ 158.30 (Cq), 148.88 (C3, C3′), 148.32 (Cq), 146.82 (Cq), 136.87 (C5, C5′), 123.21 (C6″), 128.96 (C4″), 123.18 (C6, C6′), 122.30 (C2″), 122.21(C4, C4′), 118.39 (C5″), 111.06

(C3″), 59.07 (CH_2 x 2), 56.70(CH_2), 55.84 (OCH_3), (*Figure S18*). ESI-MS (*m/z*) Calcd for: $C_{20}H_{21}N_3O_2$ 335.40, $[HL5]^+$ (*m/z* calc: 336.17), found 336.20 [M+1],(*Figure S21*). Elemental analysis: *calc:* C, 71.62; H, 6.31; N, 12.53; *found:* C, 70.55; H, 6.45; N, 12.47

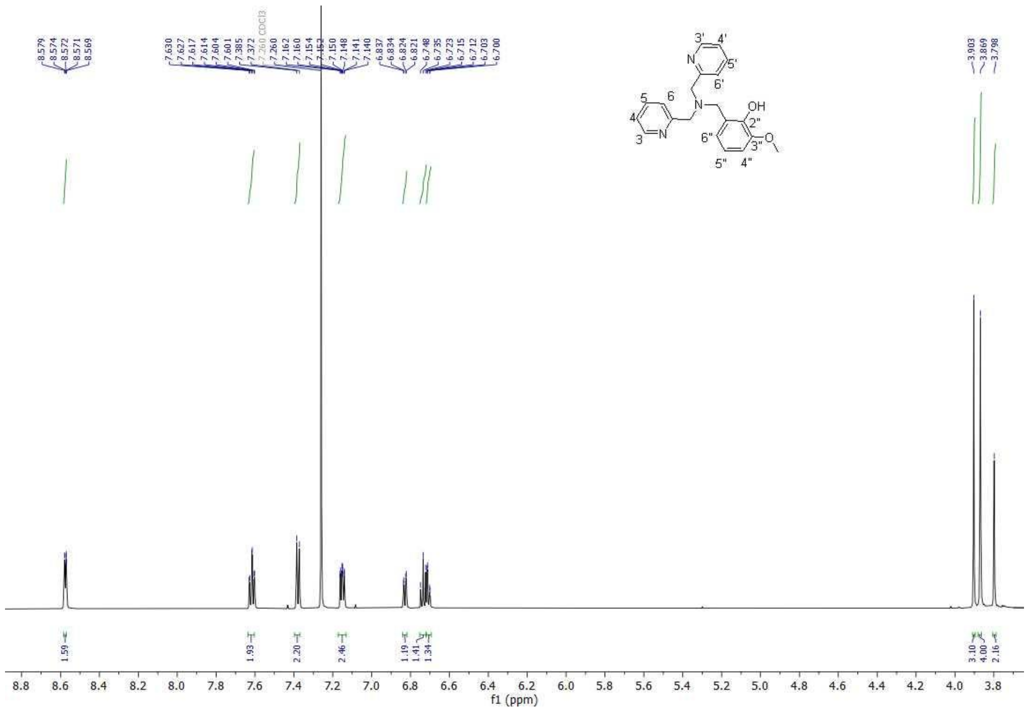

Figure S17. [1]*H NMR (CDCl₃, 600 MHz) copy of compound* ***HL5***.

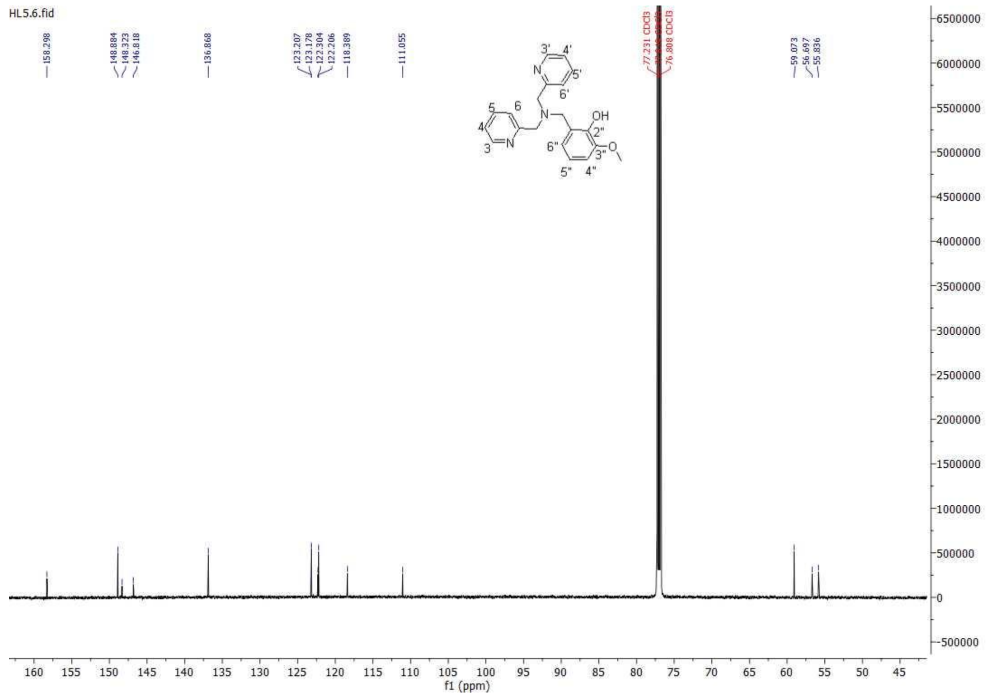

Figure S18. [13]*C NMR (CDCl₃, 150 MHz) copy of compound* ***HL5.***

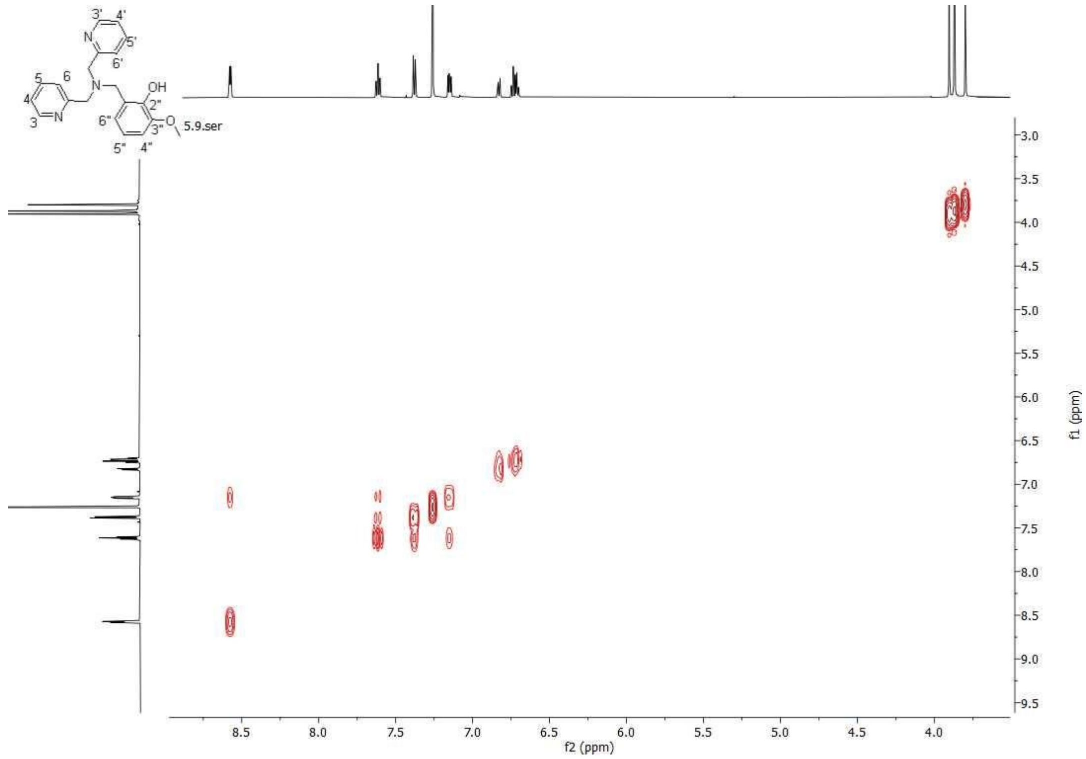

Figure S19. *^{1}H–^{1}H COSY copy of compound* ***HL5.***

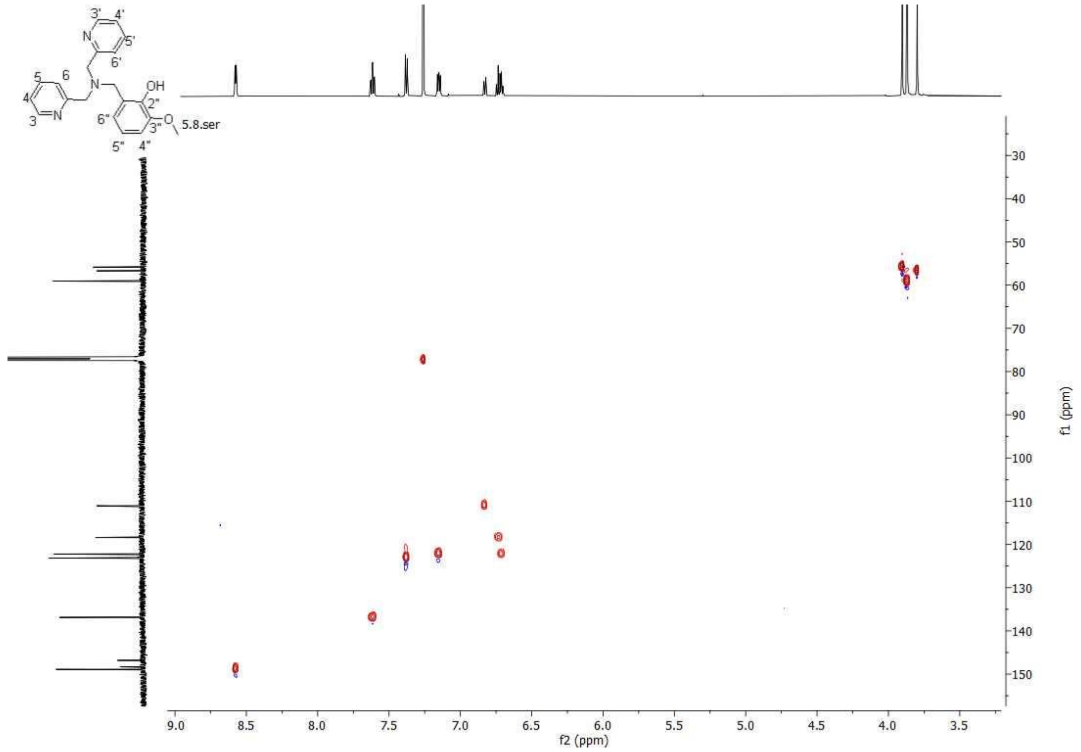

Figure S20. *^{1}H–^{13}C HSQC copy of compound* ***HL5.***

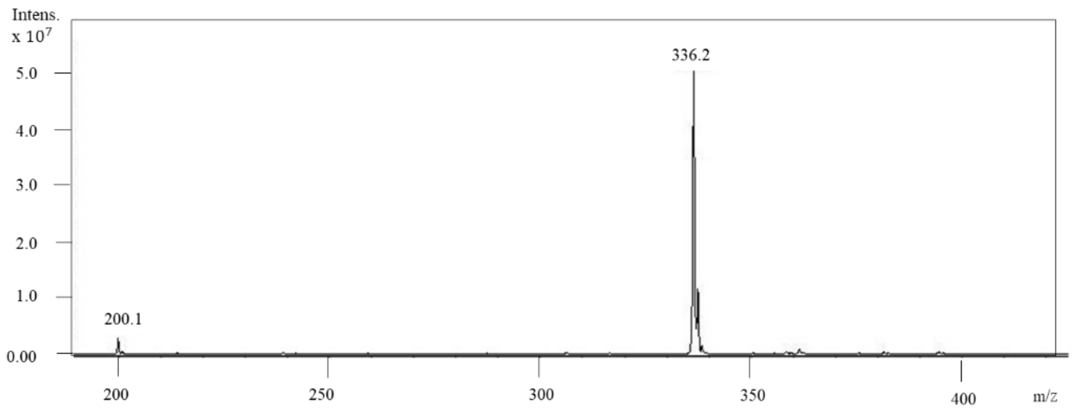

Figure S21.*ESI-MS (+) (CH$_3$CN, m/z) spectrum of compound* ***HL5.***

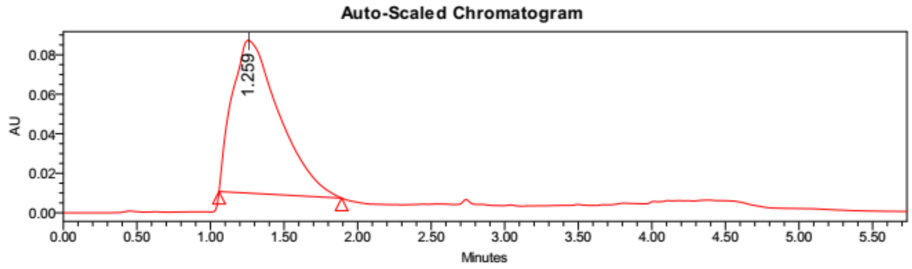

Peak Results

	Name	RT	Area	Height	% Area
1		1.259	1731872	77388	100.00

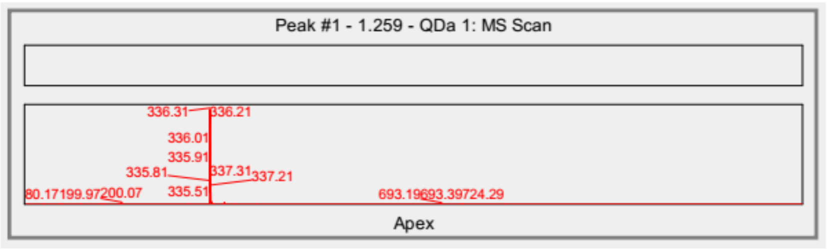

Figure S22.*UHPLC-MS chromatogram of compound* ***HL5.***

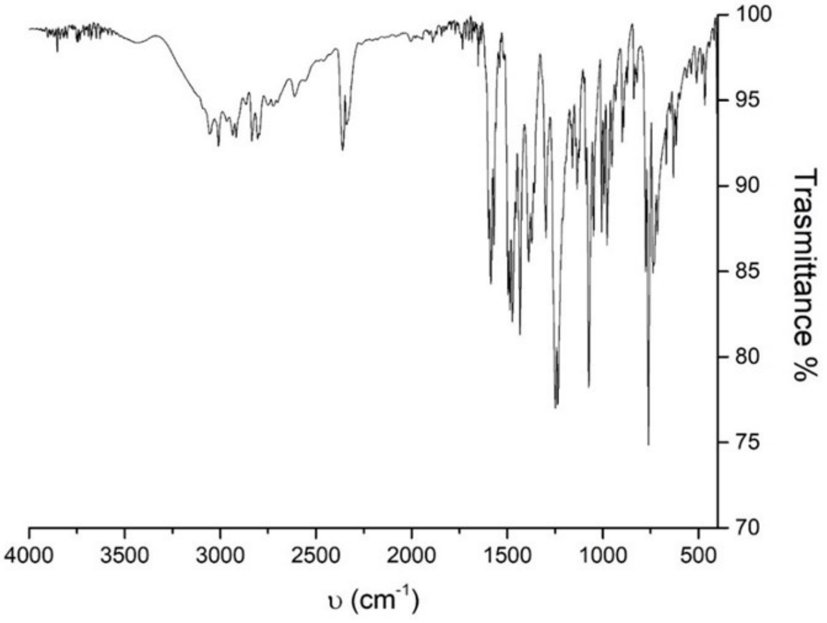

Figure S23: *FT-IR spectrum (KBr) of* ***HL5****.*

5. **Synthesis and characterization of the complexes**

Synthesis of Cu(HL1)(ClO$_4$)$_2$

The mononuclear copper complex, Cu(HL1)(ClO$_4$)$_2$ (*Figure S24*) was synthesized using HL1 (0.0905 g, 0.30 mmol) and copper perchlorate (0.107 g, 0.29 mmol) in MeOH or CH_3CN.

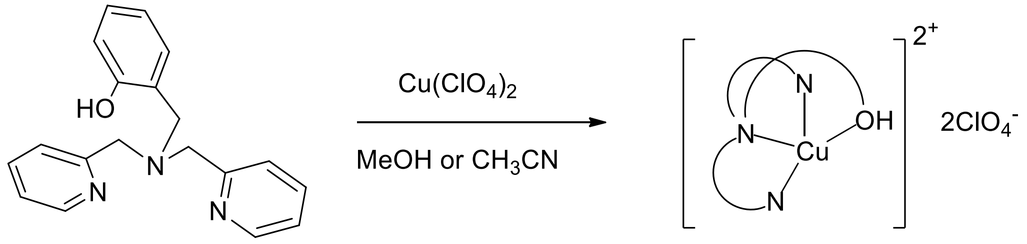

Figure S24: *Synthesis of the mononuclear complex Cu(HL1)(ClO$_4$)$_2$.*

ESI-MS (+) in CH_3CN (*Figure S25*): m/z 367.1, (calc. for [CuHL1]$^+$ 367.07)

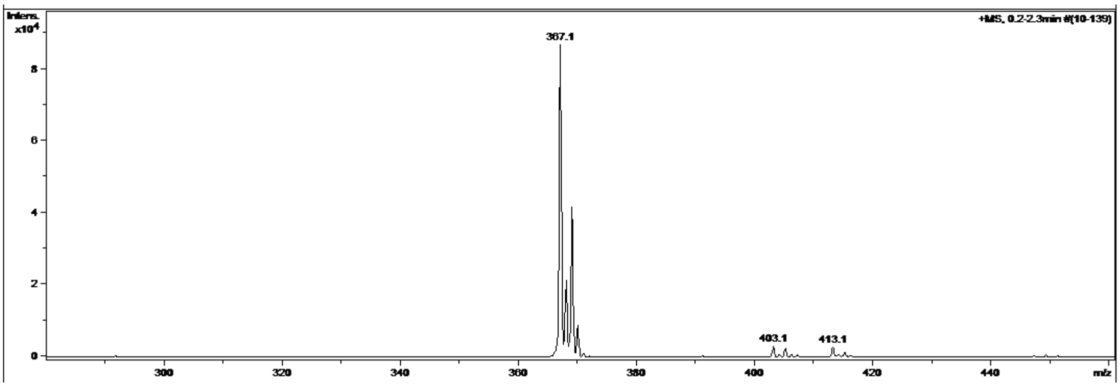

Figure S25: *ESI-MS (+) spectrum of the complex Cu(HL1)(ClO_4)$_2$ (4x10^{-6}M, CH_3CN, m/z).*

Elemental analysis (*calc* for $C_{23}H_{25}Cl_2CuN_5O_9$*:* C, 37.79; H, 3.84, N, 6.96; *found:* C, 37.25; H, 3.78; N, 7.18) indicates the presence of two water molecules in [Cu(HL1)(ClO_4)$_2$]·2H_2O

The crystal structure of the HL1 monomeric complex was determined by X-ray crystallography (*Figure S26* and Table S2), using crystals grown from acetonitrile/Et_2O at room temperature. The study revealed that the crystallization solvent occupies one of the four equatorial positions. For this reason, the crystallized complex is best formulated as [Cu(HL1)(CH_3CN)ClO_4](ClO_4)·CH_3CN (**1**). Interestingly, a literature analog by TuczeK *et al*,[5] featuring acetone as the crystallization solvent, is reported to crystallize in the *P*2$_1$/*c* space group, with very similar unit cell parameters, at 170 K, indicating the possibility, for **1**, to undergo a phase transition between room temperature and 170 K.

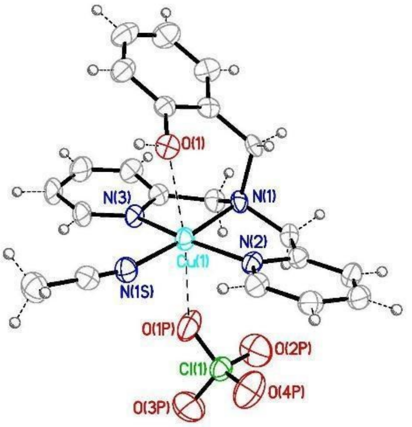

Figure S26: *Molecular structure of the [Cu(HL1)(CH_3CN)ClO_4]$^+$ cation in* **1**, *with selected atomic labels (ellipsoids drawn at the 30% probability level).*

[5] Wendt, F; Rolff, M; Thimm, W.; Nather, C.; Tuczek, F., *Z. Anorg. Allg. Chem.*, **2013**, *639* (14), 2502-2509.

Table S2: Selected *bond lengths (Å) and angles (°) for [Cu(HL1)(CH3CN)ClO4](ClO4)·CH3CN (**1**).*

Cu1-N1	2.032(12)
Cu1-N2	1.988(13)
Cu1-N3	1.980(12)
Cu1-N1S	1.989(13)
Cu1-O1	2.466(10)
Cu1-O1P	2.580(10)
N1-Cu1-N2	83.6(5)
N1-Cu1-N3	86.5(5)
N1-Cu1- N1S	174.0(6)
N1-Cu1-O1	87.7(4)
N1-Cu1-O1P	93.0(4)
N2-Cu1-N3	166.0(5)
N2-Cu1-N1S	97.0(6)
N2-Cu1-O1	98.8(4)
N2-Cu1-O1P	90.2(4)
N3-Cu1-N1S	96.1(5)
N3-Cu1-O1	87.0(5)
N3-Cu1-O1P	84.2(4)
N1S-Cu1-O1	86.3(5)
N1S-Cu1-O1P	92.9(5)
O1-Cu1-O1P	171.1(4)

Synthesis of $Cu_2(L1)_2(ClO_4)_2$

The dinuclear complex $Cu_2(L1)_2(ClO_4)_2$ (*Figure S27*) was synthesized by dissolving HL1 (0.158 g, 0.52 mmol) in 10 mL of methanol (MeOH), and adding it to a solution of $Cu(ClO_4)_2$ (0.198 g, 0.53 mmol) in 5 mL of MeOH. Triethylamine (Et_3N, 80 μL) was then added to the mixture, which was stirred for 30 minutes at room temperature. The reaction mixture was subsequently heated at 65 °C for 15 minutes. During heating, small aliquots of acetonitrile (CH_3CN) were added to redissolve any precipitate that formed. After cooling to room temperature, the solution was stored in the freezer. After two weeks, blue crystals were isolated by filtration and washed with cold MeOH, followed by diethyl ether.

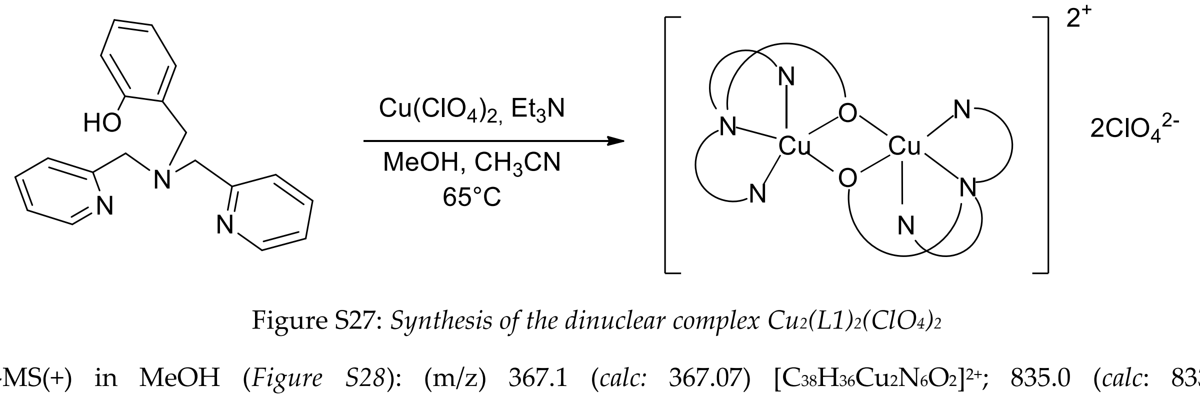

Figure S27: *Synthesis of the dinuclear complex $Cu_2(L1)_2(ClO_4)_2$*

ESI-MS(+) in MeOH (*Figure S28*): (m/z) 367.1 (*calc:* 367.07) $[C_{38}H_{36}Cu_2N_6O_2]^{2+}$; 835.0 (*calc*: 833.1) $[C_{38}H_{36}ClCu_2N_6O_6]^{+}$.

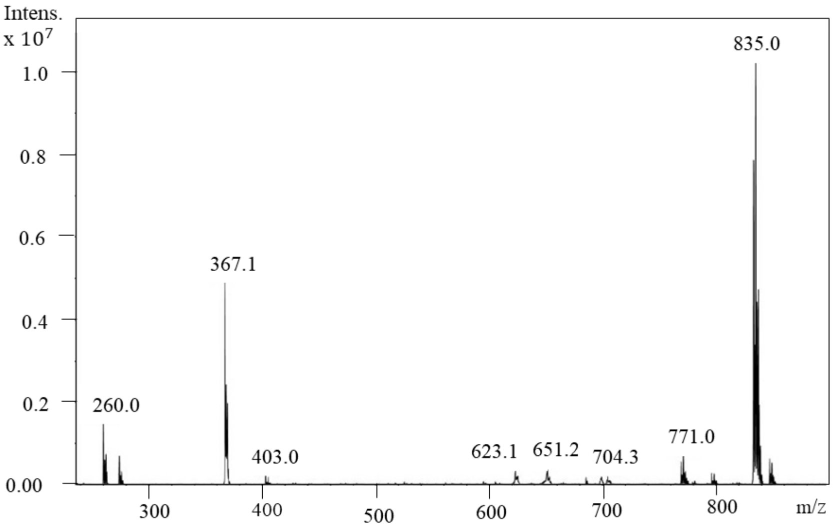

Figure S28: *ESI-MS (+) spectrum of the complex* $Cu_2(L1)_2(ClO_4)_2$ *(10^{-5}M, MeOH).*

Elemental analysis: (*calc.* for $C_{38}H_{38}Cl_2Cu_2N_6O_{10}$ C, 48.83; H, 3.88; N, 8.99; *found***:** 48.87; H, 3.97; N, 9.09), that confirms the presence of the dinuclear complex $Cu(L1)_2(ClO_4)_2$.

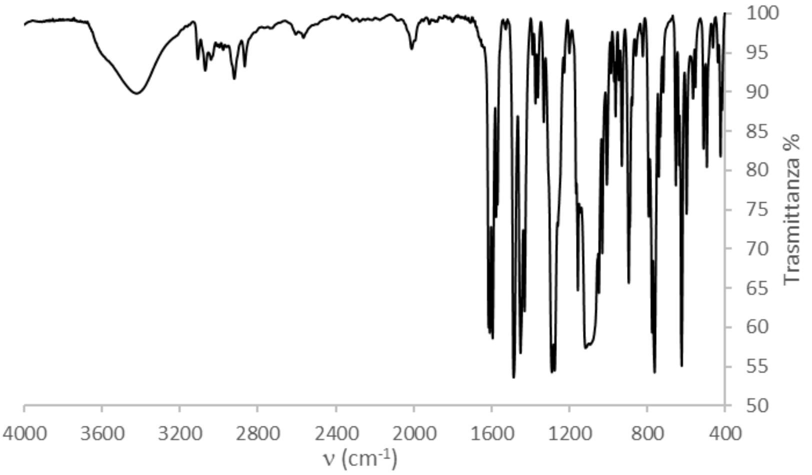

Figure S29: *FT-IR spectrum (KBr) of the complex* $Cu_2(L1)_2(ClO_4)_2$.

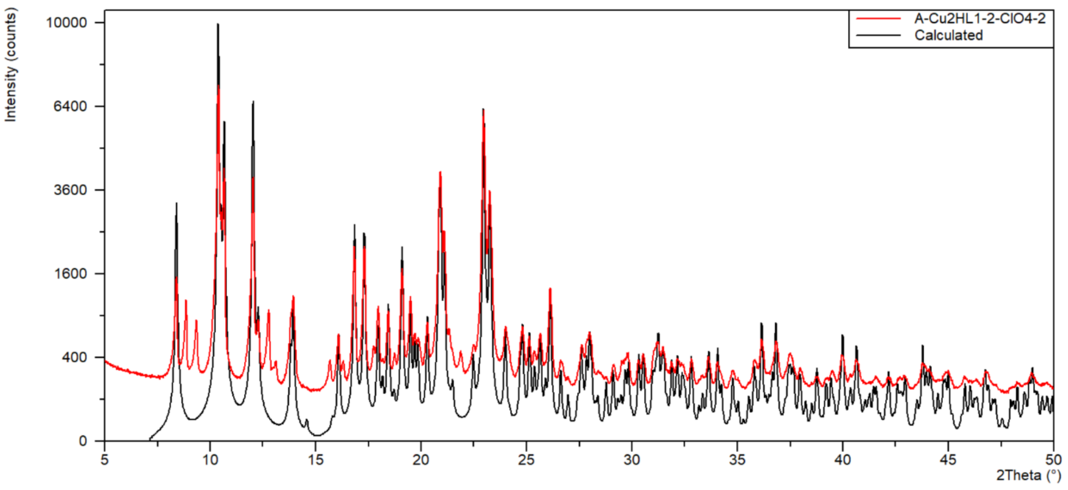

Figure S30: *The image shows the calculated powder X-ray diffraction (PXRD) pattern of $Cu_2HL1_2(ClO_4)_2$ (in black, CCDC ref. code TENZIV01),*[6] *overlaid with the experimental PXRD pattern (in red). The PXRD analysis confirms that the main phase present corresponds to the $Cu_2HL1_2(ClO_4)_2$ complex. Additional peaks are observed at 8.9°, 9.4°, 12.8°, 13.1°, 15.7°, 16.4° and 21.8° which are attributed to a different crystalline phase.*

Synthesis of Cu(HL5)(ClO_4)$_2$

The mononuclear copper complex Cu(HL5)(ClO_4)$_2$ (*Figure S31)* was synthesized using HL5 (0.0908 g, 0.27 mmol) and copper perchlorate (0.105 g, 0.28 mmol) in MeOH or CH_3CN.

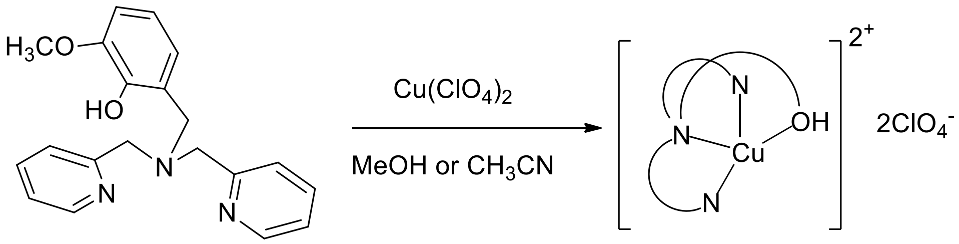

Figure S31: *Synthesis of the mononuclear complex Cu(HL5)(ClO_4)$_2$.*

ESI-MS(+) in CH_3CN (*Figure S32*): m/z 397.2 (*calc.* $[C_{20}H_{20}CuN_3O_2]^+$ 397.08) .

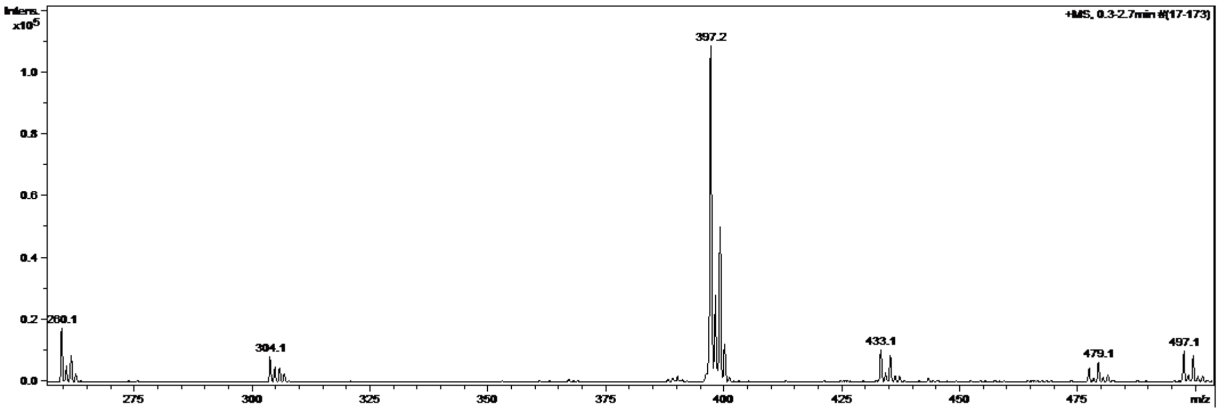

Figure S32: *ESI-MS (+) spectrum of the complex Cu(HL5)(ClO_4)$_2$ (4x10^{-5}M, CH_3CN).*

[6] Kani Y., Ohba S., Ito S., Nishida Y. *Acta Crystallogr. C.* **2000**, C56, e201.

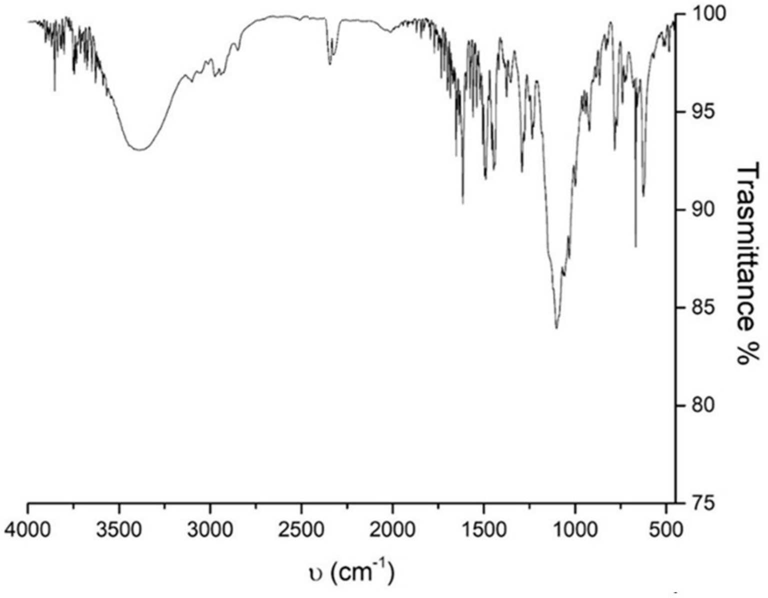

Figure S33: *FT-IR spectrum (KBr) of the complex Cu(HL5)(ClO₄)₂*

Elemental analysis: (*calc:* C, 39.01; H, 3.76; N, 6.82; *found:* C, 39.06; H, 4.00; N, 6.86) confirmed the presence of one molecule of water

The monomeric Cu(II) complex with the HL5 ligand was characterized by X-ray diffraction using crystals grown either from CH_3CN/Et_2O or $MeOH/Et_2O$. Crystals grown from CH_3CN/Et_2O were stable at room temperature, while those from $MeOH/Et_2O$ required low-temperature analysis (153 K) to prevent decomposition. In both cases, the crystallization solvent enters the coordination sphere of the Cu(II) ions, occupying one of the four equatorial positions. The resulting complexes are thus best formulated as $[Cu(HL5)(CH_3CN)ClO_4](ClO_4)$ (**2**) (*Figure S34a*) and $[Cu(HL5)(MeOH)ClO_4](ClO_4)$ (**3**) (*Figure S34b)*. See Table S3 for selected bond lengths and angles.

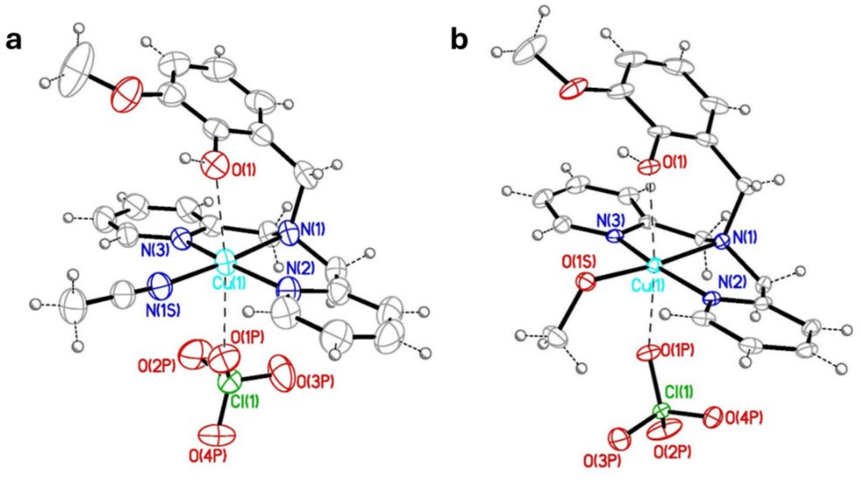

Figure S34: *Molecular structure of a)* $[Cu(HL5)(CH_3CN)ClO_4]^+$ *and b)* $[Cu(HL5)(CH_3OH)ClO_4]^+$ *cations in* **2** *and* **3**, *respectively, with selected atomic labels (ellipsoids drawn at the 30% probability level). The positional disorder of the perchlorate oxygen atoms is not shown, for clarity.*

Table S3: Selected *bond lengths (Å) and angles (°) for [Cu(HL5)(CH$_3$CN)ClO$_4$](ClO$_4$)* (**2**) *and [Cu(HL5)(MeOH)ClO$_4$](ClO$_4$)* (**3**). *Note: "S1" is N1S for* **2** *and O1S for* **3**.

	**2**	**3**
Cu1-N1	2.020(3)	2.027(4)
Cu1-N2	1.981(3)	1.968(4)
Cu1-N3	1.977(3)	1.979(4)
Cu1-S1	1.981(3)	1.985(3)
Cu1-O1	2.487(2)	2.470(3)
Cu1-O1P	2.576(3)	2.504(3)
N1-Cu1-N2	83.26(12)	83.70(15)
N1-Cu1-N3	84.13(11)	84.58(15)
N1-Cu1-S1	175.08(12)	173.06(14)
N1-Cu1-O1	89.30(10)	88.12(13)
N1-Cu1-O1P	96.23(11)	92.35(13)
N2-Cu1-N3	165.16(12)	167.15(16)
N2-Cu1-S1	96.30(13)	95.50(15)
N2-Cu1-O1	96.78(11)	98.61(12)
N2-Cu1-O1P	87.21(11)	91.74(13)
N3-Cu1-S1	96.93(12)	96.77(16)
N3-Cu1-O1	90.85(10)	86.24(12)
N3-Cu1-O1P	86.41(10)	83.49(13)
S1-Cu1-O1	85.89(11)	85.18(12)
S1-Cu1-O1P	88.64(11)	94.57(13)
O1-Cu1-O1P	173.48(9)	169.62(11)

Synthesis of Cu$_2$(L5)$_2$(ClO$_4$)$_2$

The HL5-dinuclear complex, Cu$_2$(L5)$_2$(ClO$_4$)$_2$ (*Figure S35*) was synthesized by dissolving HL5 (52.6 mg, 0.15 mmol) in 5 ml of MeOH and adding it to Cu(ClO$_4$)$_2$ (60.4 mg, 0.16 mmol in 2 ml of MeOH). Et$_3$N (27 µl) was added and the reaction was stirred for 30 min. Then, the reaction flask was immersed in the oil bath at 65 °C for 15 min and CH$_3$CN was added in small aliquots to redissolve the formed precipitate. After this step, the flask was cooled, stored in a freezer and green crystals were obtained after two weeks, filtered and washed with MeOH and diethyl ether.

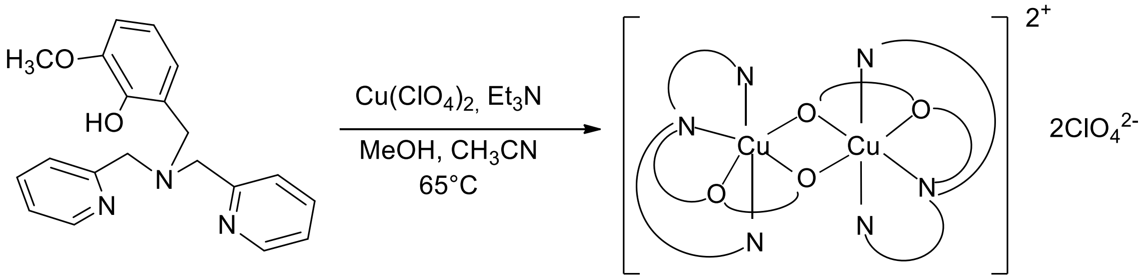

Figure S35: *Synthesis of the dinuclear complex Cu$_2$(L5)$_2$(ClO$_4$).*

ESI-MS(+) in CH$_3$CN (*Figure S35*): m/z 397.2 (*calc.* for [$C_{40}H_{40}Cu_2N_6O_4$]$^{2+}$ 397.09); m/z 895.0 (*calc.* for [$C_{40}H_{40}ClCu_2N_6O_8$]$^{+}$ 895.12).

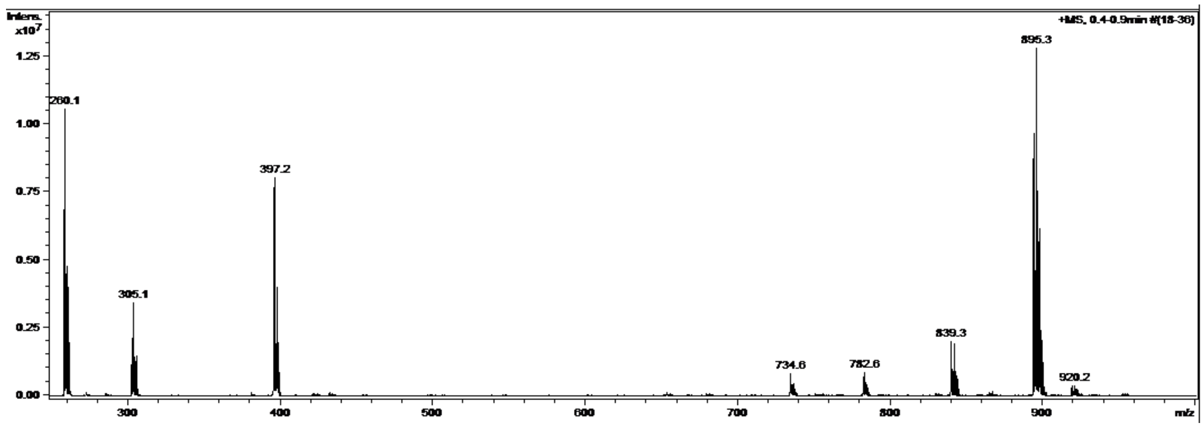

Figure S36: *ESI-MS (+) spectrum of the complex $Cu_2(L5)_2(ClO_4)_2$ (10^{-5} M, CH_3CN).*

Elemental analysis: *calc*. for $Cu_2(L5)_2(ClO_4)_2$ C, 48.30; H, 4.05; N, 8.45; *found:* 48.14; H, 4.15; N, 8.66.

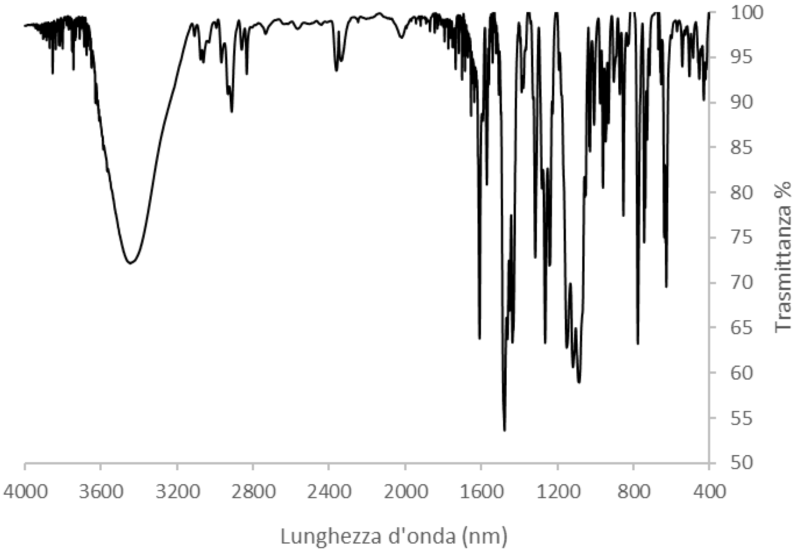

Figure S37: *FT-IR spectrum (KBr) of the complex $Cu_2(L5)_2(ClO_4)_2$*

6. **Chelating ability**

The coordination ability of HL1 and HL5 was evaluated by titrating the ligands with Cu(II) and monitoring the UV-Vis spectral changes. Two ligands concentrations (0.1 mM and 1.5 mM) were used to observe $\pi \rightarrow \pi^*$ and d-d transitions, respectively. The solutions of the ligand were obtained from a stock solution in MeOH. A quartz cuvette, with 1 cm optical path was filled with 2.5 mL of the ligand solution, then aliquots of 2.5 µL of copper (II) solution were added (0.1 eq of Cu^{2+}, for each addition). After each addition, the absorbance variation, by UV-Vis scans was followed from 200 nm to 800 nm. Both ligands showed increased absorbance (hyperchromic effect) $\pi \rightarrow \pi^*$ band and shape change in spectra. HL1 also exhibits blue shift and two isosbestic points (*Figures S38, S39*), indicating an equilibrium between species, during copper(II) addition. At 1.5 mM, both ligands showed an increase in d-d band intensity confirming complex formation.

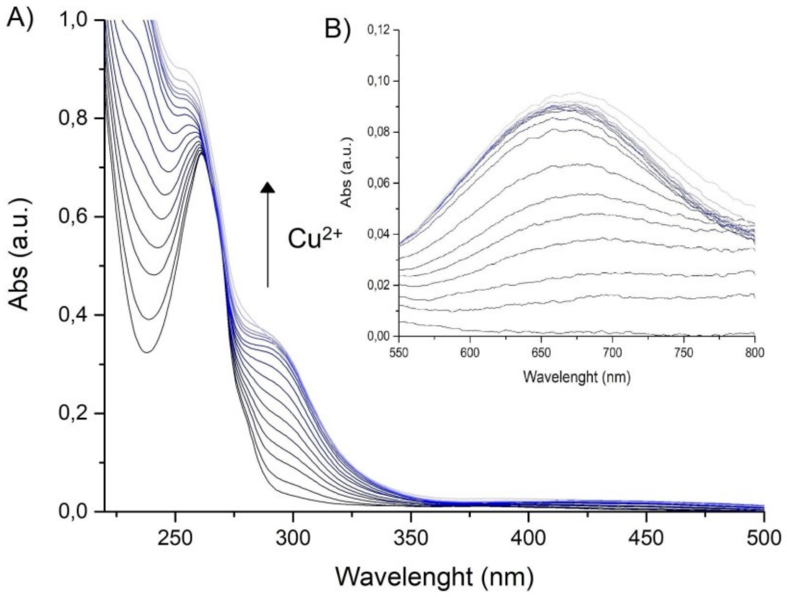

Figure S38: *UV-Vis spectra collected during HL1 titration: spectra A) shows* π→π* band, *B) d→d band. The titration was carried out using the following concentration: 0.01M Cu(II) in water and 0.1 mM of the ligand, for the π→π* band, and 0.15M Cu(II) in water and 1.5mM of the ligand.*

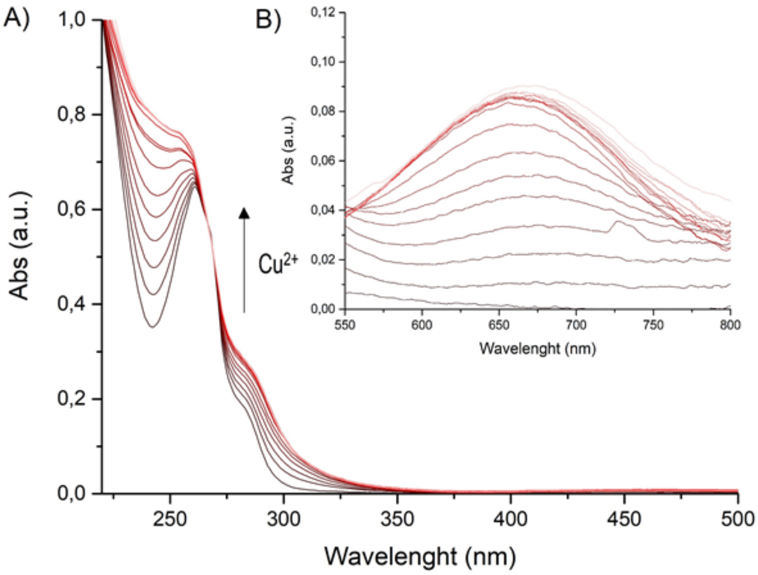

Figure S39: *UV-Vis spectra of HL5 titration: spectra A) shows* π→π* band, *B) d→d band. The titration was carried out using the following concentration: 0.01M Cu(II) in water and 0.1 mM of the ligand, for the π→π* band, and 0.15M Cu(II) in water and 1.5mM of the ligand. The solutions of the ligand were obtained from a stock solution in MeOH.*

The graph *"Abs at λ_{max} vs Cu(II) equivalent"* (*Figure S40*) highlights the formation of 1:1 complexes for both ligands. There is a progressive increase in the absorbance at the maximum absorption (d→d), until reaching the plateau, when the ratio between Cu(II) and the ligands is 0.9:1. The difference with respect to the expected ratio, 1:1, is likely due to the occurrence of different protonation states for the ligands and to the presence of the organic solvent.

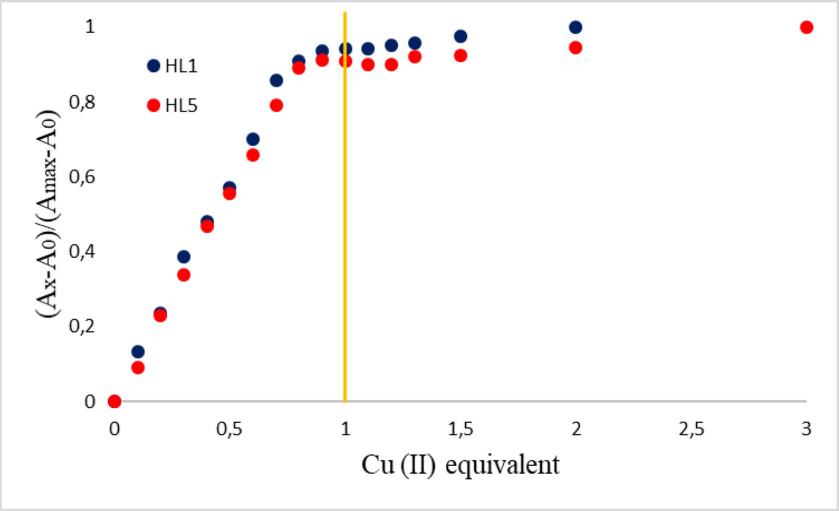

Figure S40: *Relative absorbance variation (λ_{max} of d→d band) vs. Cu(II) equivalents, obtained from the titration of HL1 (blue) and HL5 (red).*

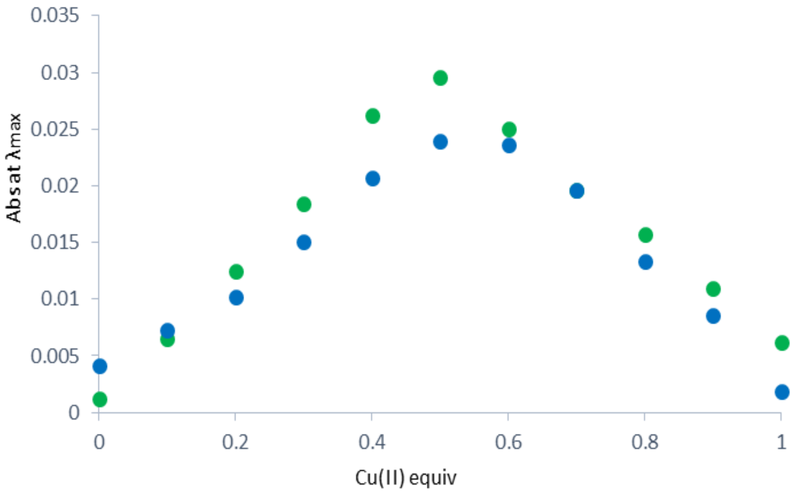

Figure S41: *Job's Plot constructed using the wavelengths of maximum absorption of the d–d bands generated by the formation of the copper complex with: HL1 (blue), HL5 (green). Conditions used: 0.75 mM solutions of ligand in water and 0.75 mM Cu(II) in water. For HL1 and HL5, which are poorly soluble in water, the aqueous solutions were prepared starting from stock solutions in methanol.*

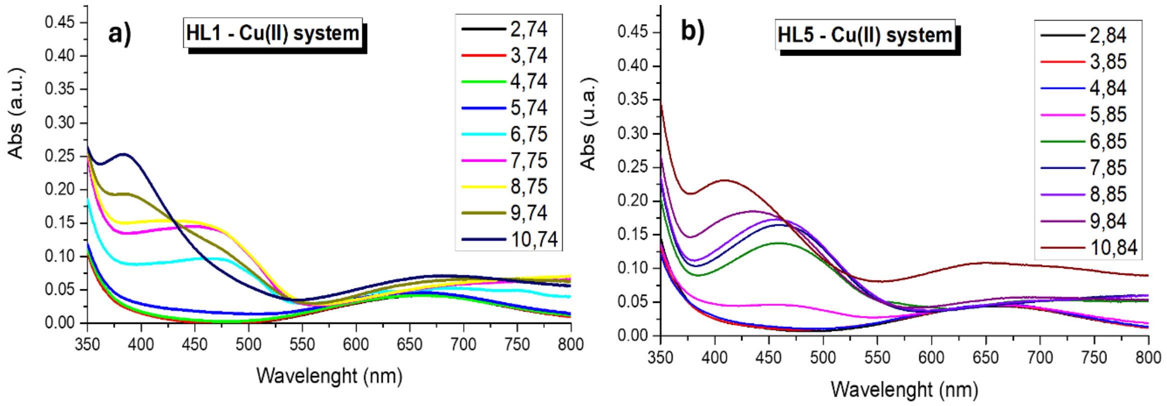

Figure S42: *pH-dependent absorption spectra of the a) HL1-Cu(II) and b) HL5-Cu(II) systems; [HL1]=1mM and [HL5]=0.6 mM; a 5% DMSO solution (v/v) with water containing HCl/KCl.*

7. Cyclic Voltammetry

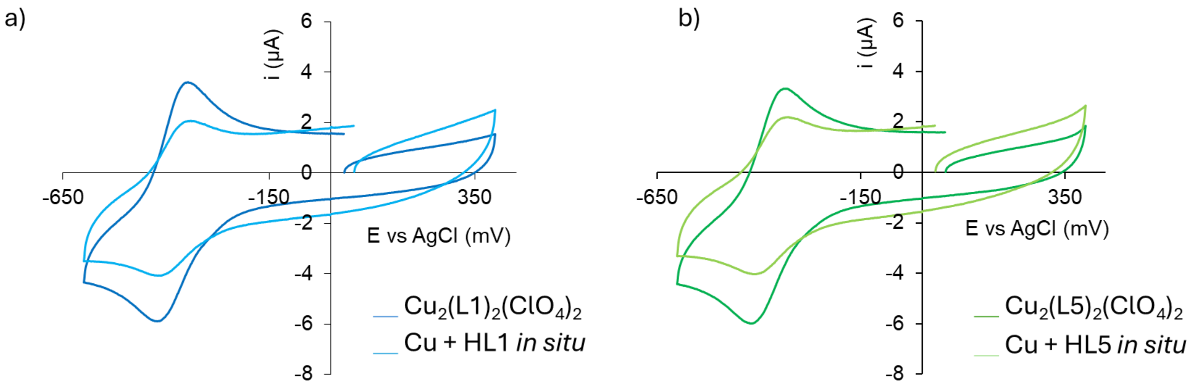

Figure S43: *Cyclic voltammograms of $Cu_2L1_2(ClO_4)_2$ (blue) and $Cu_2L5_2(ClO_4)_2$ (green) and comparison with the corresponding mononuclear complexes generated in situ (light blue and light green), recorded at scan rate 50 mV/s [$Cu_2L5_2(ClO_4)_2$] = 0.25 mM in PBS 10 mM, NaCl 100 mM, pH 7.4.*

8. Catalytic activity

To monitor the CAT-like activity, a 25 mL vial was used as a reactor, connected to a pressure transducer and a septum for the injection.

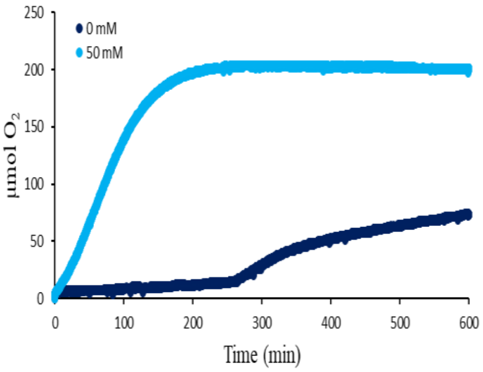

a)

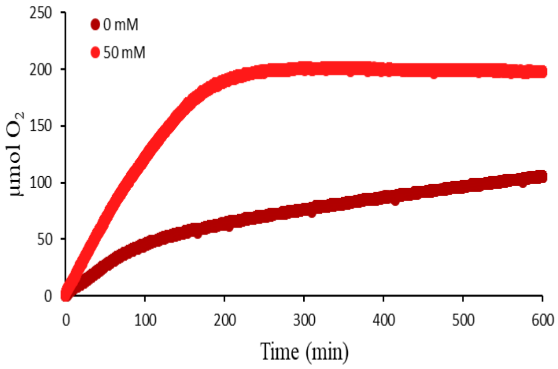

b)

Figure S44: *O_2 production over time, generated from the hydrogen peroxide dismutation. a) trend obtained for preformed complexes with HL1 (blue) and in presence of 50mM KBr (light blue); b) trend obtained for the preformed complex with HL5 (red) and in presence of 50mM KBr (light red).*

To monitor the spectral changes during the H_2O_2 dismutation, the samples were prepared in the same conditions, and monitored by a Varian Cary 50 instrument in 3 mL quartz cuvettes (1 cm optical path). Spectra were recorded at preset intervals, providing a time-resolved view of the catalytic process.

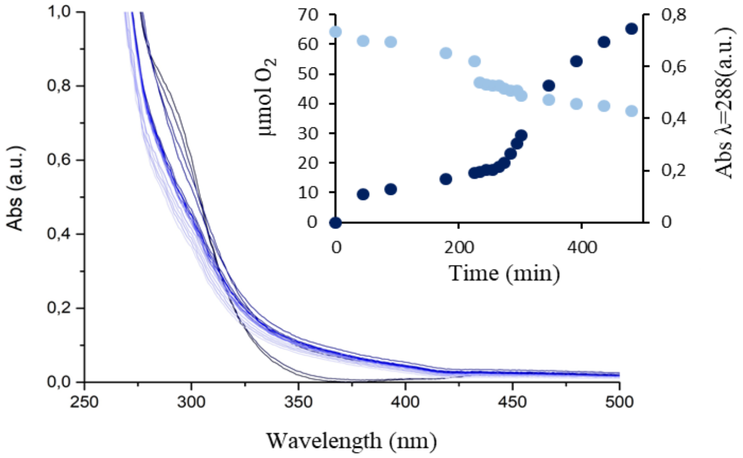

Figure S45: *UV-Vis spectra of $Cu(HL1)(ClO_4)_2$ at time 0 (blue) and after 8 h (light blue). The colours variation represents an increment of 45 min from t=0 to t=225 min, of 10 min from t=225 to t= 305 min and 45 min from t= 305 to t=485 min. Conditions: $[Cu(HL1)(ClO_4)_2]$= 200 µM, $[H_2O_2]$=10mM in BBS 50 mM pH=7.8. Zoom: temporal trends compared to O_2 (blue) and Abs (light blue) on the maximum absorption at 293 nm.*

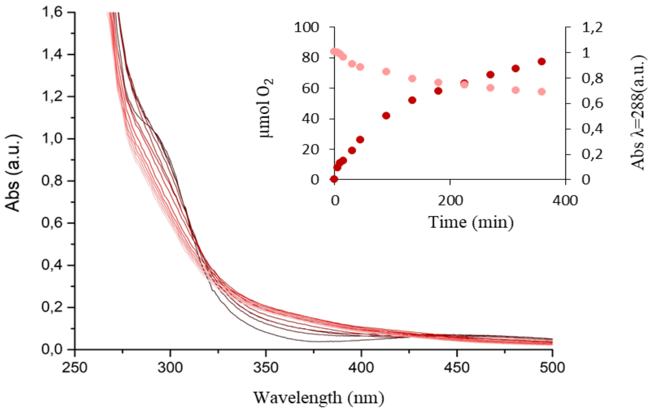

Figure S46: *UV-Vis spectra of Cu(HL5)(ClO$_4$)$_2$ at time 0 (red) and after 7 h (light red). The colours variation represents an increment of 5 min from t=0 to t=15 min, of 15 min from t=15 to t= 45 min and 45 min from t= 45 to t=360 min. Conditions: [Cu(HL5)(ClO$_4$)$_2$]= 200 uM, [H$_2$O$_2$]=10mM in BBS 50mM pH=7.8. Zoom: temporal trends compared to O$_2$ (red) and Abs (light red) on the maximum absorption at 294 nm.*

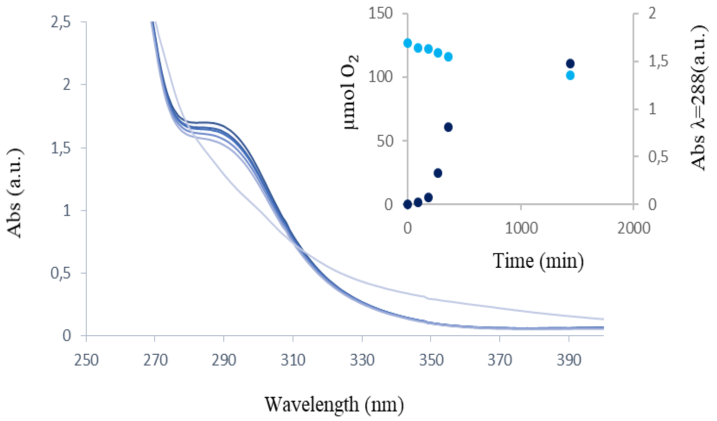

Figure S47: *UV-Vis spectra of Cu$_2$(L1)$_2$(ClO$_4$)$_2$ at time 0 (blue) and after 24 hours (light blue). Every change of colors represents a temporal increase of 90 minutes. Conditions: [Cu$_2$(L1)$_2$(ClO$_4$)$_2$] = 200 µM, [H$_2$O$_2$] = 10 mM in BBS 50 mM, pH = 7.8. Zoom: temporal trends compared to O$_2$ (blue) and Abs (light blue) on the maximum absorption at 288 nm.*

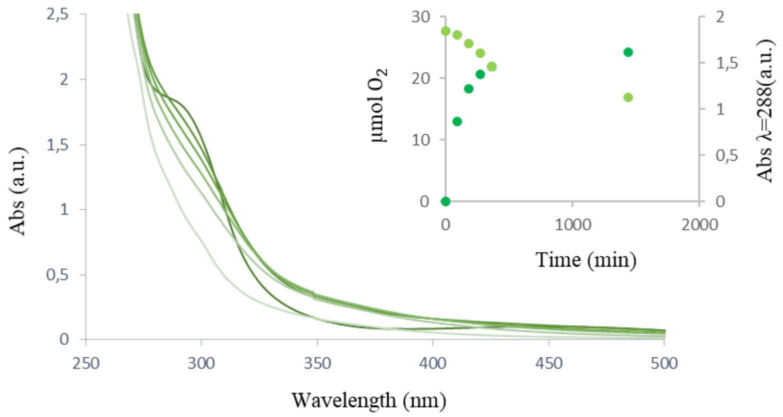

Figure S48: *UV-Vis spectra of Cu$_2$(L5)$_2$(ClO$_4$)$_2$ at time 0 (dark green) and after 24 hours (light green). Every change of colours represents a temporal increase of 90 minutes. Conditions: [Cu$_2$(L5)$_2$(ClO$_4$)$_2$] = 200 µM, [H$_2$O$_2$] = 10 mM in BBS 50 mM, pH = 7.8. Zoom: temporal trends compared to O2 (dark green) and Abs (light green) on the maximum absorption at 288 nm.*

Peroxidase activity was evaluated via UV-Vis spectroscopy by monitoring the time-dependent absorbance changes of oxidized substrates in the presence of H_2O_2 and copper complexes. Two substrates were used: morin and o-phenylenediamine (OPD).

For morin, the oxidative degradation (Figure S49) was evidenced by a decrease in absorbance at 390 nm. Measurements were performed in 50 mM PBS (0.1 M NaCl, pH 7.8) at 25 °C using a 1 cm quartz cuvette. Conditions included: Catalyst: 3.2 µM (monomeric) and 1.6 µM (dimeric), H_2O_2: 10 mM and morin: 0.055 mM. The reaction kinetics were monitored over 18 hours with spectra collected every 20 minutes (*Figures S50–S53*).

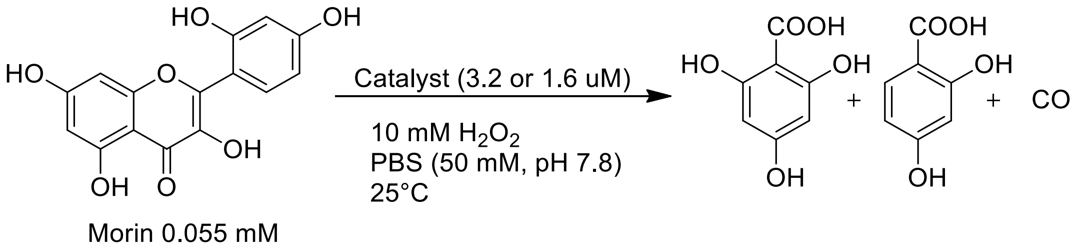

Figure S49: *Oxidative degradation of morin and the conditions used in the experiment.*

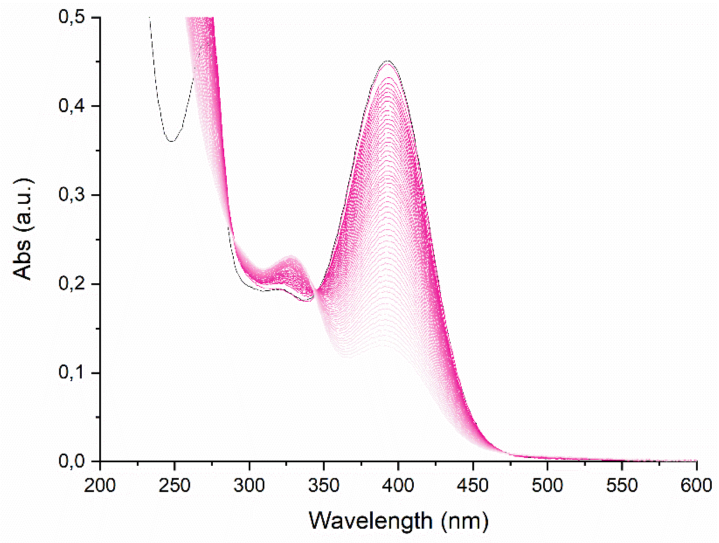

Figure S50: *Absorbance variation of the morine, with the complex Cu(HL1)(ClO$_4$)$_2$. The black line is at t=0, while the light pink line is at t=18h. The concentrations used were: PBS 50mM (0.1 M NaCl, pH=7.8),3.2 µM of Cu(HL1)(ClO$_4$)$_2$, 10 mM of H_2O_2 and 0.055 mM of morin.*

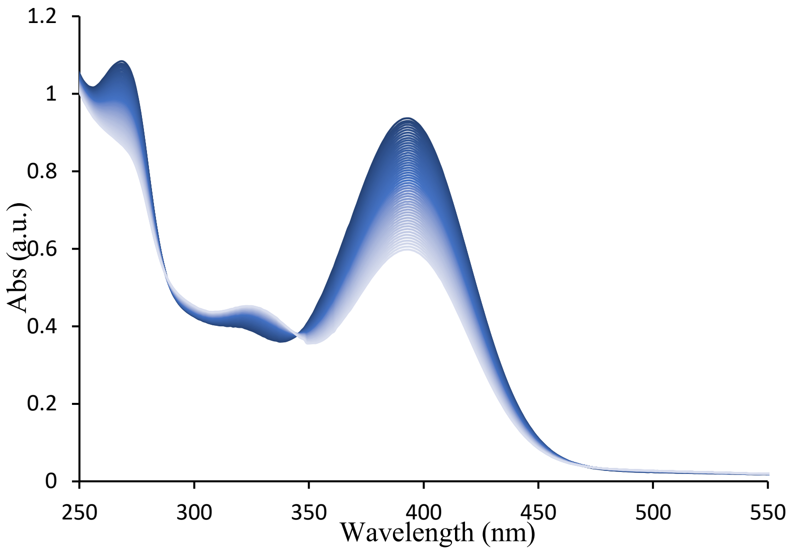

Figure S51: *Absorbance variation of the morin, with the complex $Cu_2(L1)_2(ClO_4)_2$. The dark blue line is at t=0, while the light blue line is at t=18h. The concentrations used were PBS 50mM (0.1 M NaCl, pH=7.8), 1.6 µM of $Cu_2(L1)_2(ClO_4)_2$, 10 mM of H_2O_2 and 0.055 mM of morin.*

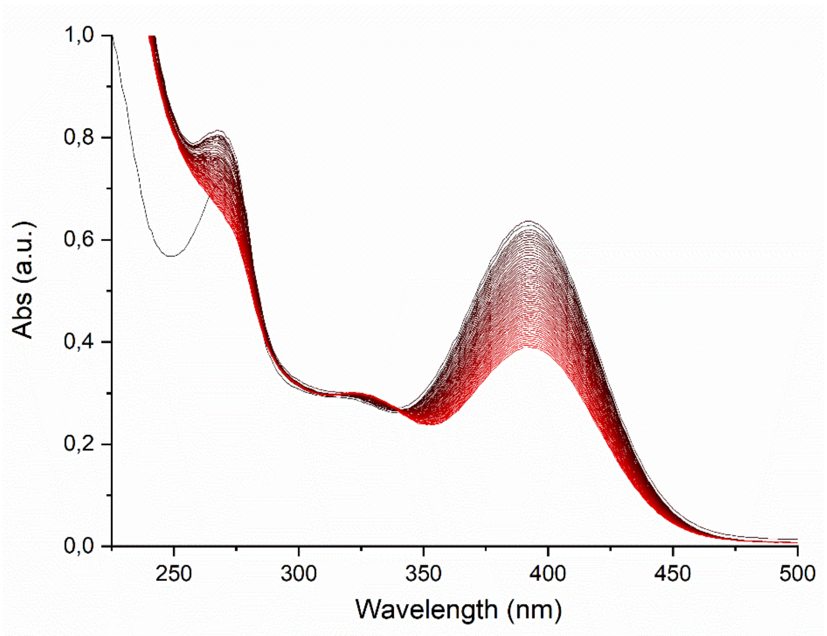

Figure S52: *Absorbance variation of the morin, with the complex $Cu(HL5)(ClO_4)_2$. The black line is at t=0, while the light red line is at t=18h. The concentrations used were: PBS 50mM (0.1 M NaCl, pH=7.8),3.2 µM of $Cu(HL5)(ClO_4)_2$, 10 mM of H_2O_2 and 0.055 mM of morin.*

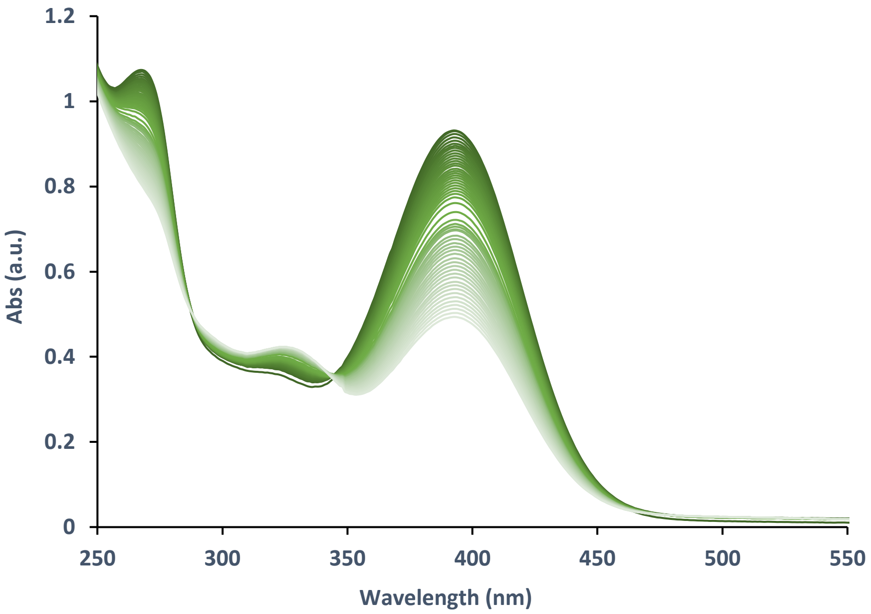

Figure S53: *Absorbance variation of the morin, with the complex $Cu_2(L5)_2(ClO_4)_2$. The dark green line is at t=0, while the light green line is at t=18h. The concentrations used were: PBS 50mM (0.1 M NaCl, pH=7.8), 1.6 µM of $Cu_2(L5)_2(ClO_4)_2$, 10 mM of H_2O_2 and 0.055 mM of morin.*

The second substrate tested was o-phenylenediamine (OPD), which oxidizes to 2,3-diaminophenazine (DAP), a colored and fluorescent product. (*Figure S54*) The reaction was monitored by the increase in absorbance at 420 nm, corresponding to DAP formation.

Experiments were performed in 50 mM phosphate buffer (0.1 M NaCl, pH 7.8) at 25 °C using a 1 cm quartz cuvette. Conditions included: Catalyst: 3.2 µM (monomeric) and 1.6 µM (dimeric), H_2O_2: 0.024 M and OPD: 0.32 mM. Kinetic data were collected over 18 hours at 20-minute intervals (F*igures S55–S58*).

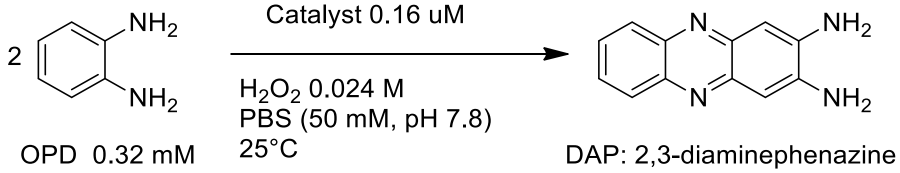

Figure S54: *Oxidative degradation of OPD and the conditions used in the experiment.*

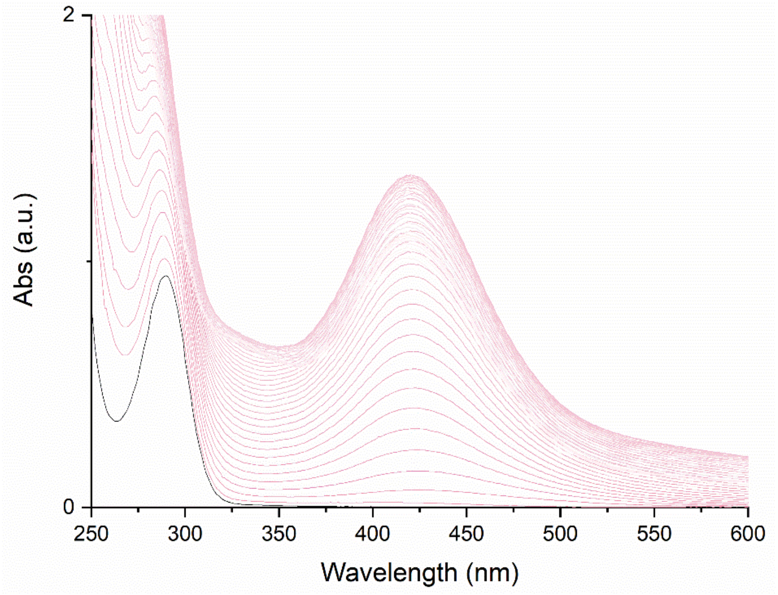

Figure S55: *Increase in the absorbance at 420 nm of DAP in the presence of the complex $Cu(HL1)(ClO_4)_2$. The black line is at t=0, while the light pink line is at t=18h. The concentrations used were: PBS 50mM (0.1 M NaCl, pH=7.8), 3.2 µM of $Cu(HL1)(ClO_4)_2$, 0.024 M of H_2O_2 and 0.32 mM of OPD.*

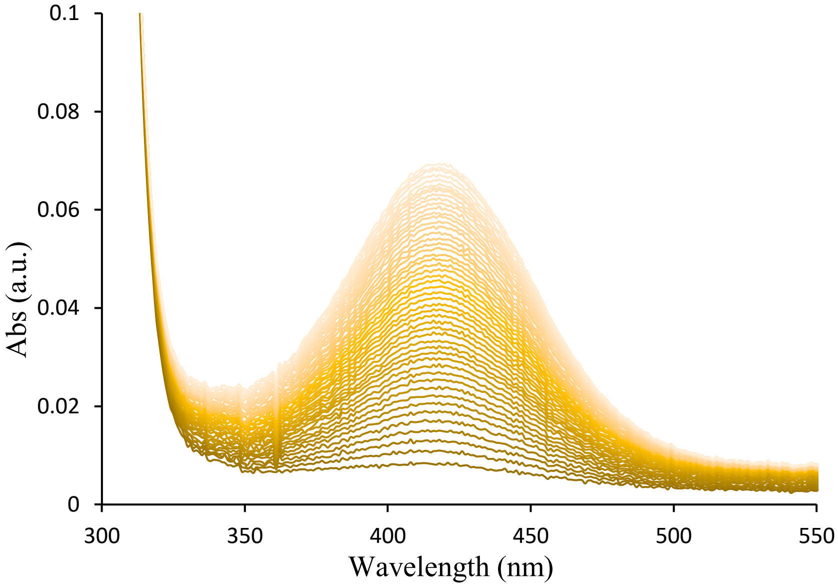

Figure S56: *Increase in the absorbance at 420 nm of DAP in the presence of the complex $Cu_2(L1)_2(ClO_4)_2$ The dark yellow line is at t=0, while the light-yellow line is at t=18h. The concentrations used were: PBS 50mM (0.1 M NaCl, pH=7.^{86} µM of $Cu_2(L1)_2(ClO_4)_2$, 0.024 M of H_2O_2 and 0.32 mM of OPD.*

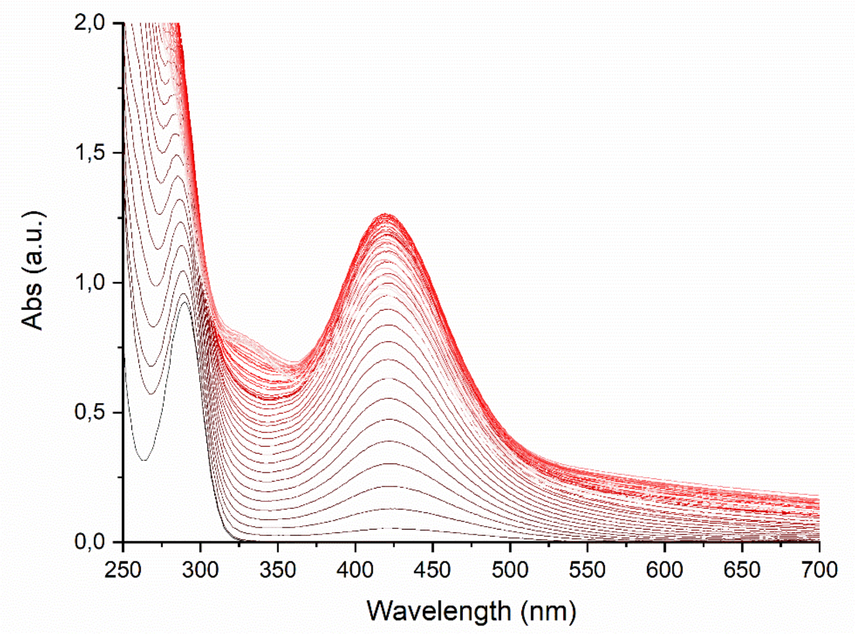

Figure S57: *Increase in the absorbance at 420 nm of DAP in the presence of the complex Cu(HL5)(ClO$_4$)$_2$. The black line is at t=0, while the light red line is at t=18h. The concentrations used were PBS 50mM (0.1 M NaCl, pH=7.8), 3.2 µM of Cu(HL5)(ClO$_4$)$_2$, 0.024 M of H$_2$O$_2$ and 0.32 mM of OPD.*

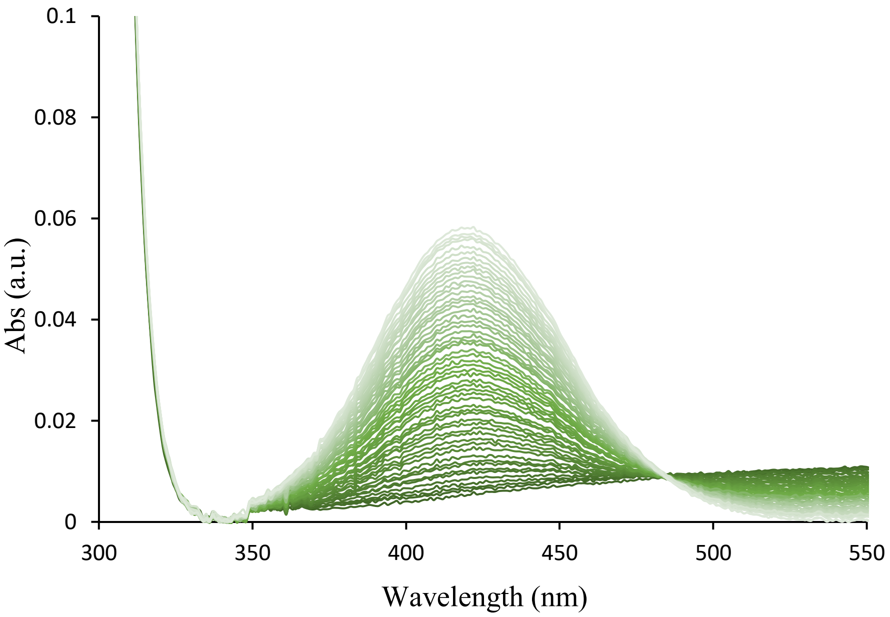

Figure S58: *Increase in the absorbance at 420 nm of DAP in the presence of the complex Cu$_2$(L5)$_2$(ClO$_4$)$_2$ The dark green line is at t=0, while the light-green line is at t=18h. The concentrations used were: PBS 50mM (0.1 M NaCl, pH=7.8), 1.6 µM of Cu$_2$(L5)$_2$(ClO$_4$)$_2$, 0.024 M of H$_2$O$_2$ and 0.32 mM of OPD.*
